# A protective role for N-acylphosphatidylethanolamine phospholipase D in 6-OHDA-induced neurodegeneration

**DOI:** 10.1038/s41598-019-51799-1

**Published:** 2019-11-04

**Authors:** Francesca Palese, Silvia Pontis, Natalia Realini, Daniele Piomelli

**Affiliations:** 1 Department of Drug Discovery and Development, Fondazione Istituto Italiano di Tecnologia, via Morego 30, 16163 Genoa, Italy; 2 Departments of Anatomy and Neurobiology and Biological Chemistry, University of California, Irvine, CA 92697-4625 USA

**Keywords:** Parkinson's disease, Cell death in the nervous system

## Abstract

N-acylphosphatidylethanolamine phospholipase D (NAPE-PLD) catalyzes the cleavage of membrane NAPEs into bioactive fatty-acid ethanolamides (FAEs). Along with this precursor role, NAPEs might also serve autonomous signaling functions. Here, we report that injections of 6-hydroxydopamine (6-OHDA) into the mouse striatum cause a local increase in NAPE and FAE levels, which precedes neuronal cell death. NAPE, but not FAE, accumulation is enhanced in mice lacking NAPE-PLD, which display a substantial reduction in 6-OHDA-induced neurotoxicity, as shown by increased survival of substantia nigra dopamine neurons, integrity of striatal dopaminergic fibers, and striatal dopamine metabolite content. Reduced damage is accompanied by attenuation of the motor response evoked by apomorphine. Furthermore, NAPE-PLD silencing protects cathecolamine-producing SH-SY5Y cells from 6-OHDA-induced reactive oxygen species formation, caspase-3 activation and death. Mechanistic studies in mice suggest the existence of multiple molecular contributors to the neuroprotective effects of NAPE-PLD deletion, including suppression of Rac1 activity and attenuated transcription of several genes (*Cadps*, *Casp9*, *Egln1*, *Kcnj6*, *Spen*, and *Uchl1*) implicated in dopamine neuron survival and/or Parkinson’s disease. The findings point to a previously unrecognized role for NAPE-PLD in the regulation of dopamine neuron function, which may be linked to the control of NAPE homeostasis in membranes.

## Introduction

The N-acylphosphatidylethanolamines (NAPEs) are a quantitatively minor family of glycerophospholipids present in the membrane of all mammalian cells^1^. They are produced by transfer of an acyl group from the *sn*-1 position of phosphatidylcholine (PC) to the free amino group of phosphatidylethanolamine (PE), which is catalyzed by the calcium-dependent *N*-acyltransferase activity^2^ of cytosolic phospholipase A_2_ε (PLA2A4E)^3^. Since PLA2A4E is not selective with regard to the chain length or degree of unsaturation of the acyl chain transferred, the molecular composition of cellular NAPEs predominantly reflects the *sn*-1 substituent in the donor PC^4^. Consequently, the *N-*acyl groups in NAPEs are dominated by saturated or monounsaturated acyl species such as palmitate (16:0), stearate (18:0) or oleate (18:1), with a lesser contribution from polyunsaturated species such as arachidonate (20:4) and docosahexaenoate (22:6)^5,6^. NAPEs are cleaved by a selective phospholipase D, NAPE-PLD (encoded in mice by the *Napepld* gene), a membrane-associated zinc hydrolase^7,8^ that attacks the distal phosphodiester bond of NAPEs producing fatty-acid ethanolamides (FAEs) and phosphatidic acid.

The FAEs are a structurally and functionally heterogeneous class of lipid-derived mediators that include endogenous agonists for cannabinoid receptors [e.g., arachidonoylethanolamide (anandamide)], nuclear peroxisome proliferator-activated receptor type-α [e.g., oleoylethanolamide (OEA) and palmitoylethanolamide (PEA)] and ligand-activated ion channels such as TRPV-1 (e.g., OEA)^9^. The FAEs participate in a wide range of physiological and pathological processes, such as neurotransmission (anandamide)^10^, pain (anandamide, PEA)^10–12^, energy balance (OEA)^13,14^ and inflammation (PEA)^15^. The NAPEs have been primarily studied for their role as FAE precursors, but evidence indicates that they might also serve autonomous structural and signaling functions^16^. For example, biophysical experiments suggest that NAPEs may contribute to cell-membrane dynamics through a varied set of mechanisms that include membrane stabilization^17,18^, stimulation of calcium-dependent membrane fusion^19^, and consolidation of lipid raft structure^20^. Furthermore, similarly to the better known phosphoinositides^21^, the NAPEs might serve as tethers for the association of intracellular proteins to the internal facet of the lipid bilayer^22^.

Ischemic insults to the brain cause a rapid and profound elevation in NAPE levels^23–25^. Similar responses have been documented in primary cultures of brain neurons exposed to neurotoxic insults, such as high concentrations of the excitatory transmitter glutamate^26–28^. It is still unknown, however, whether damage-induced NAPE accrual plays a functional role in neurotoxicity and neurodegeneration. We have recently shown that intrastriatal injections of the dopaminergic neurotoxin 6-hydroxydopamine (6-OHDA) produce a local accumulation of *N*-acyl saturated NAPE species^29^. In the present study, we examined the impact of genetic NAPE-PLD deletion on 6-OHDA-induced neurotoxicity in mice and in the catecholamine-producing human cell line SH-SY5Y. The results suggest that complete or partial NAPE-PLD ablation enhances 6-OHDA-induced NAPE accumulation without significantly changing FAE levels, and concomitantly protects dopamine neurons from the toxic effects of 6-OHDA. We also explored potential molecular mechanisms through which NAPE-PLD ablation might protect mice from 6-OHDA-induced toxicity. A previous report has shown that exogenous NAPE application inhibits activity of the Rho family GTP-binding protein 1 (Rac1)^30^, which has been implicated in dopamine neuron survival^31,32^. Consistent with those results, we found that genetic NAPE-PLD ablation is associated with a significant reduction in bioactive GTP-bound Rac1. However, focused transcriptomic analyses revealed several additional changes in genes involved in dopamine neuron function, suggesting that a multiplicity of mechanisms might contribute to neuroprotection in NAPE-PLD-null mice.

## Results

### Effects of 6-OHDA on striatal NAPE levels

**Figure 1:**
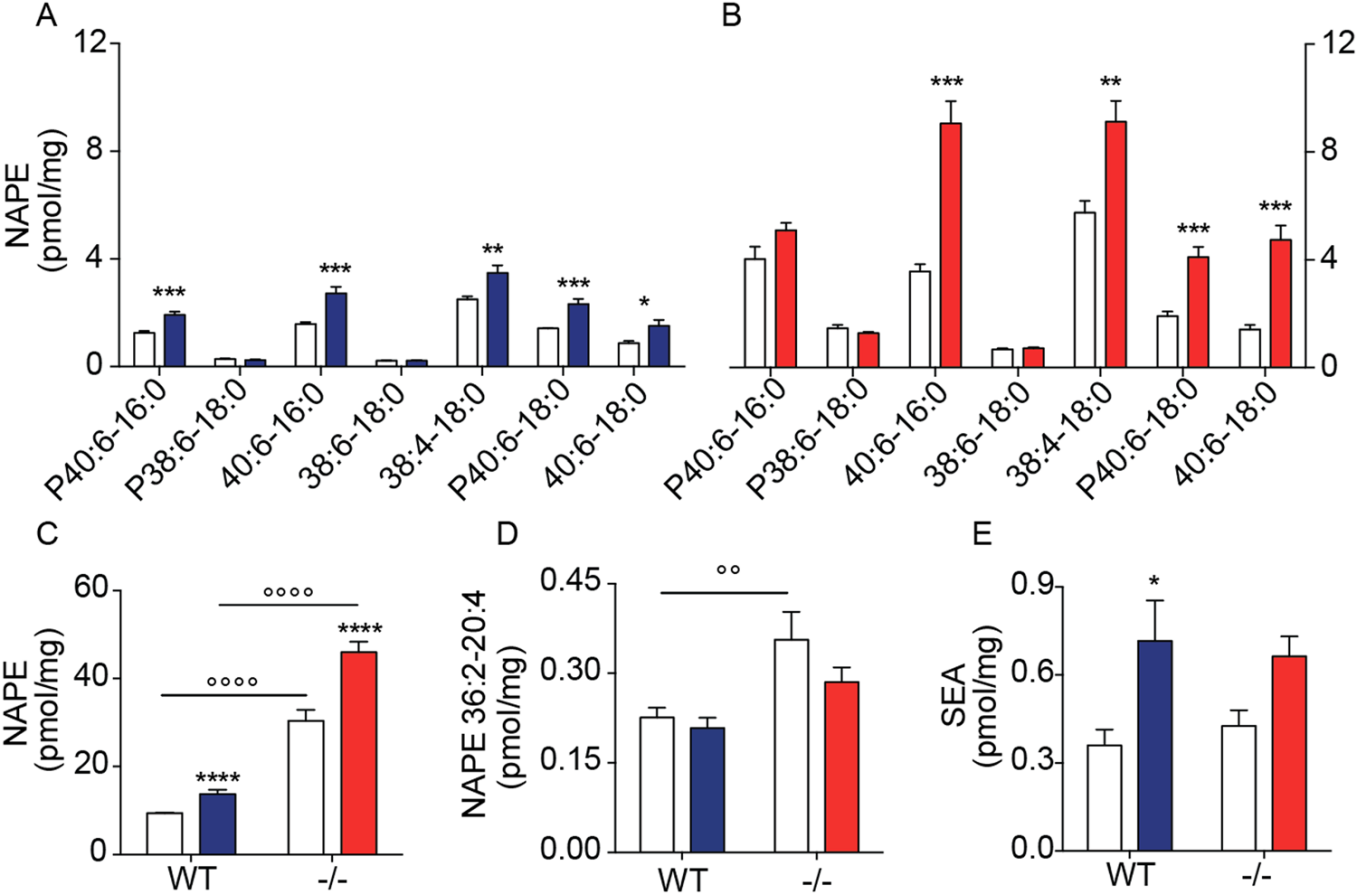
Effects of NAPE-PLD deletion on NAPE and FAE levels in mouse striatum. (**A**,**B**) Levels of individual NAPEs in dorsal striatum of (**A**) wild-type (WT) mice, and (**B**) NAPE-PLD^−/−^ mice 48 h after intrastriatal 6-OHDA administration; open bars: control side of both WT and NAPE-PLD^−/−^ mice; color-coded bars: lesioned side of WT (blue) or NAPE-PLD^−/−^ (red) mice (n = 6–7). (**C**) Total NAPE levels in striatum of WT and NAPE-PLD^−/−^ (−/−); open bars: control side for both WT and NAPE-PLD^−/−^ mice; color-coded bars: lesioned side of WT (blue) or NAPE-PLD^−/−^ (red) mice (n = 6–7). (**D**) Levels of anandamide precursor NAPE (36:2–20:4) in striatum of WT and NAPE-PLD^−/−^ mice. Same color-coding as in (**C**) (n = 6–7). (**E**) SEA levels in striatum of WT and NAPE-PLD^−/−^ mice. Same color-coding as in (**C**) (n = 6–7). *P < 0.05, **P < 0.01, ***P < 0.001, ****P < 0.0001 one-way ANOVA; °°°°P < 0.0001 two-way ANOVA, Bonferroni post hoc test.

We administered 6-OHDA unilaterally in the dorsal striatum of NAPE-PLD^−/−^ mice^33^ and their wild-type littermates, and measured NAPE content by liquid chromatography tandem mass spectrometry (LC/MS-MS) 48 h later. As previously shown^29^, 6-OHDA injections increased striatal NAPE levels in wild-type mice (Fig. 1A,C). Individual NAPEs affected by 6-OHDA treatment included 1,2 diacyl and 1-alkyl-2-acyl species that contained saturated *N*-acyl substituents, such as NAPE (40:6–16:0) and NAPE (38:4–18:0) (Fig. 1A). Larger increases in NAPE content were seen in NAPE-PLD^−/−^ mice, in which baseline NAPE content was higher than controls (Fig. 1B,C). Notably, the levels of NAPE (36:2–20:4), a precursor for the endocannabinoid anandamide, were greater in NAPE-PLD^−/−^ mice than in wild-type animals, but were not affected by 6-OHDA (Fig. 1D). Accumulation of the saturated FAE stearoylethanolamide (18:0) was increased by 6-OHDA injections, but not significantly affected by NAPE-PLD deletion (Fig. 1E). The results confirm previous studies indicating that (*i*) 6-OHDA injections increase the levels of *N*-saturated NAPE species in mouse striatum^29^, and (*ii*) NAPE-PLD activity controls NAPE levels *in vivo*^33–35^.

### NAPE-PLD deletion protects dopaminergic neurons from 6-OHDA-induced toxicity

**Figure 2:**
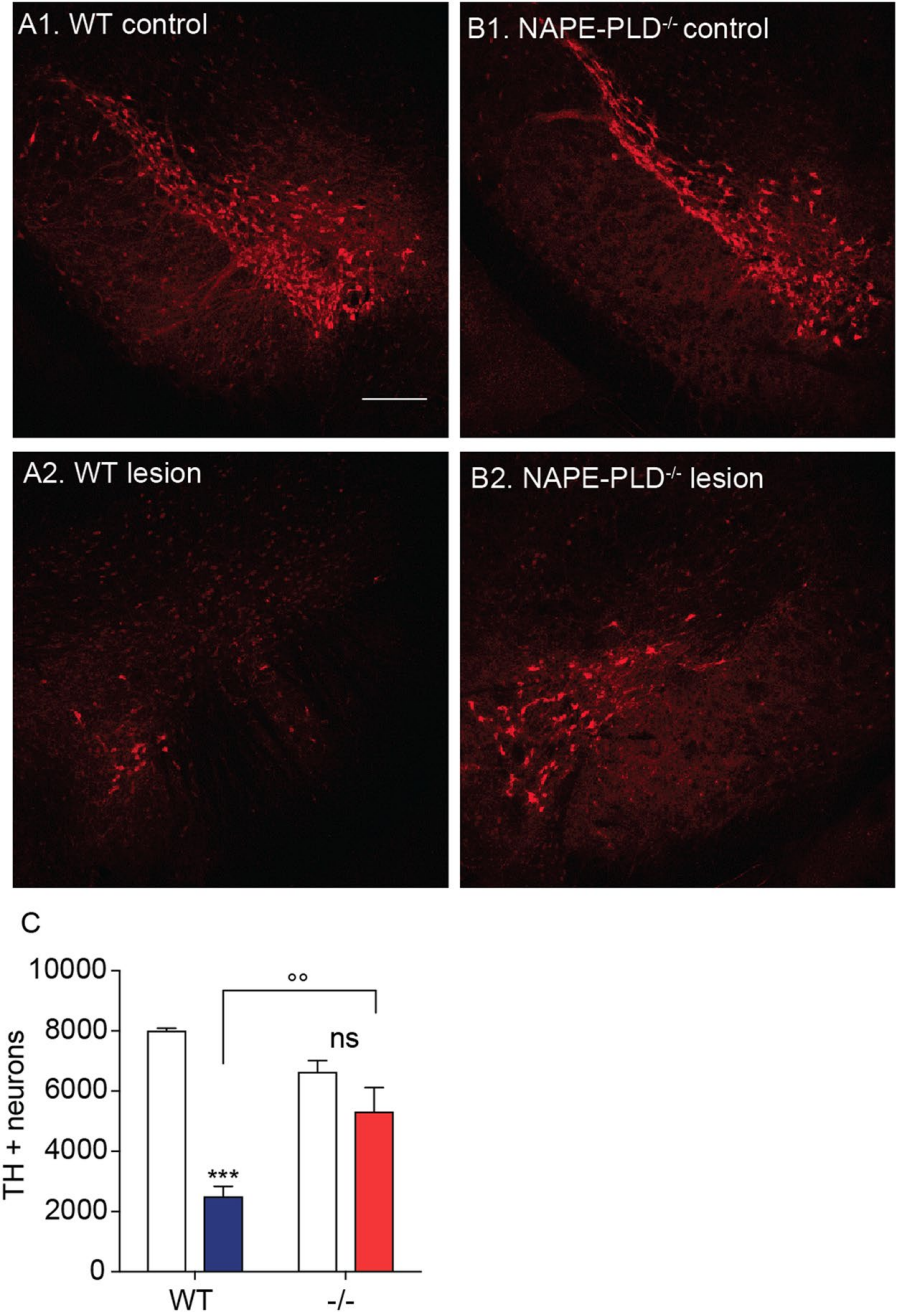
Effects of NAPE-PLD deletion on 6-OHDA-induced neurotoxicity in mouse SN. (**A**,**B**) Immunofluorescence images for TH in tissue sections of SN pars compacta: (**A1**,**B1**) control and (**A2**,**B2**) lesioned (ipsilateral and contralateral, respectively, to the 6-OHDA injection site) of wild-type (**A1–2**) or NAPE-PLD^−/−^ (**B1-2**) mice. Scale bar 200 µm. (**C**) Stereological count of TH^+^ neurons in SN pars compacta of WT and NAPE-PLD^−/−^ mice. Open bars: control side of both WT and NAPE-PLD^−/−^ mice; color-coded bars: lesioned side of WT (blue) or NAPE-PLD^−/−^ (red) mice (n = 3–4). ***P < 0.001 compared to intact contralateral side; °°P < 0.01 compared to lesioned side of wild-type mice, two-way ANOVA, Bonferroni post hoc test.

**Figure 3:**
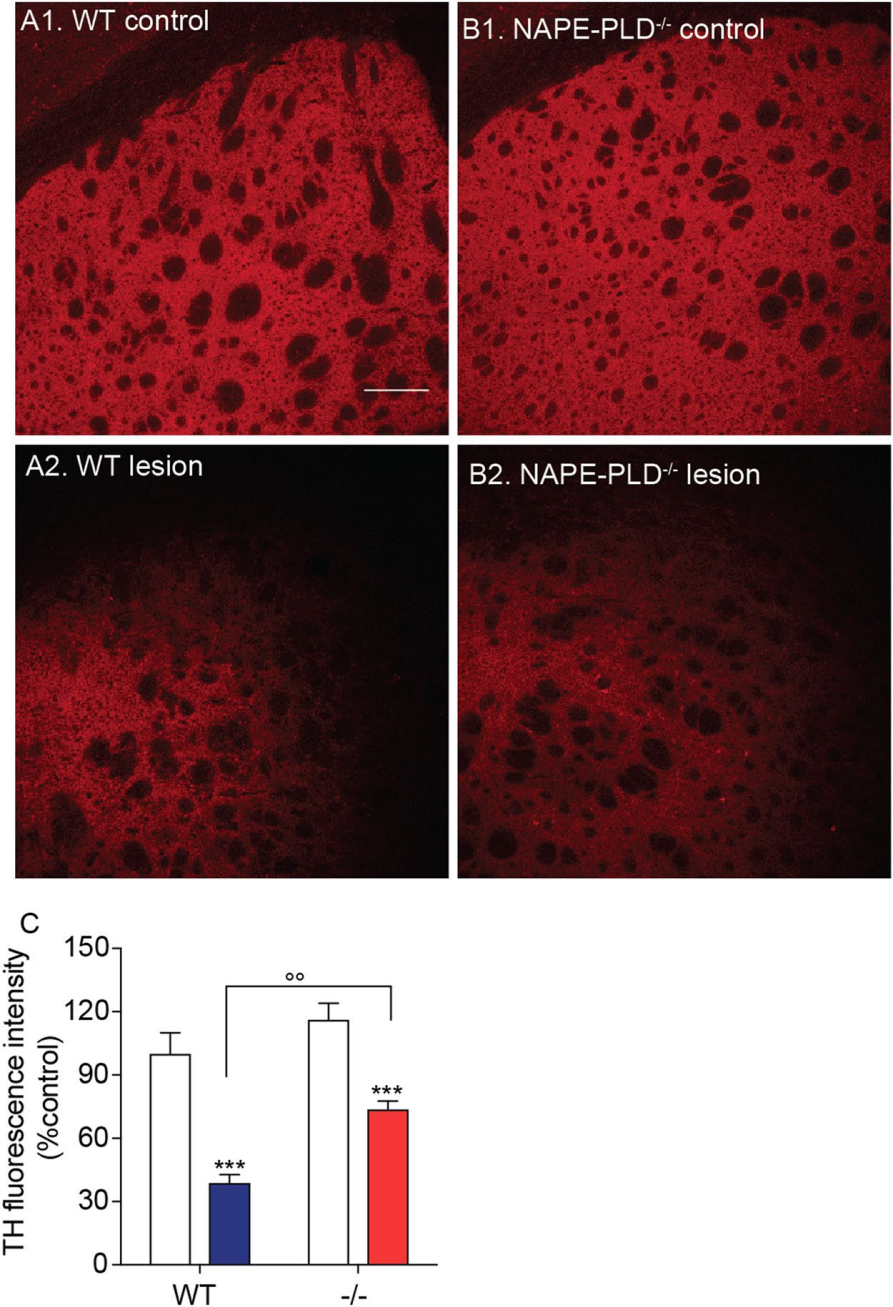
Effects of NAPE-PLD deletion on 6-OHDA-induced neurotoxicity in mouse striatum. (**A**,**B**) Immunofluorescence images for TH in tissue sections of dorsal striatum: control or lesioned (contralateral to the 6-OHDA injection site) of wild-type (**A1**, **2** respectively) or NAPE-PLD^−/−^ (**B1**, **2** respectively) mice. Scale bar, 200 µm. (**C**) Densitometric quantification of TH staining. Open bars: control side of both WT and NAPE-PLD^−/−^ mice; color-coded bars: lesioned side of WT (blue) or NAPE-PLD^−/−^ (red) mice (n = 6 mice, with 1–7 measurements each). ***P < 0.001 compared to intact contralateral side; °°P < 0.01 compared to lesioned side of wild-type mice, two-way ANOVA, Bonferroni post hoc test compared to intact side.

**Figure 4:**
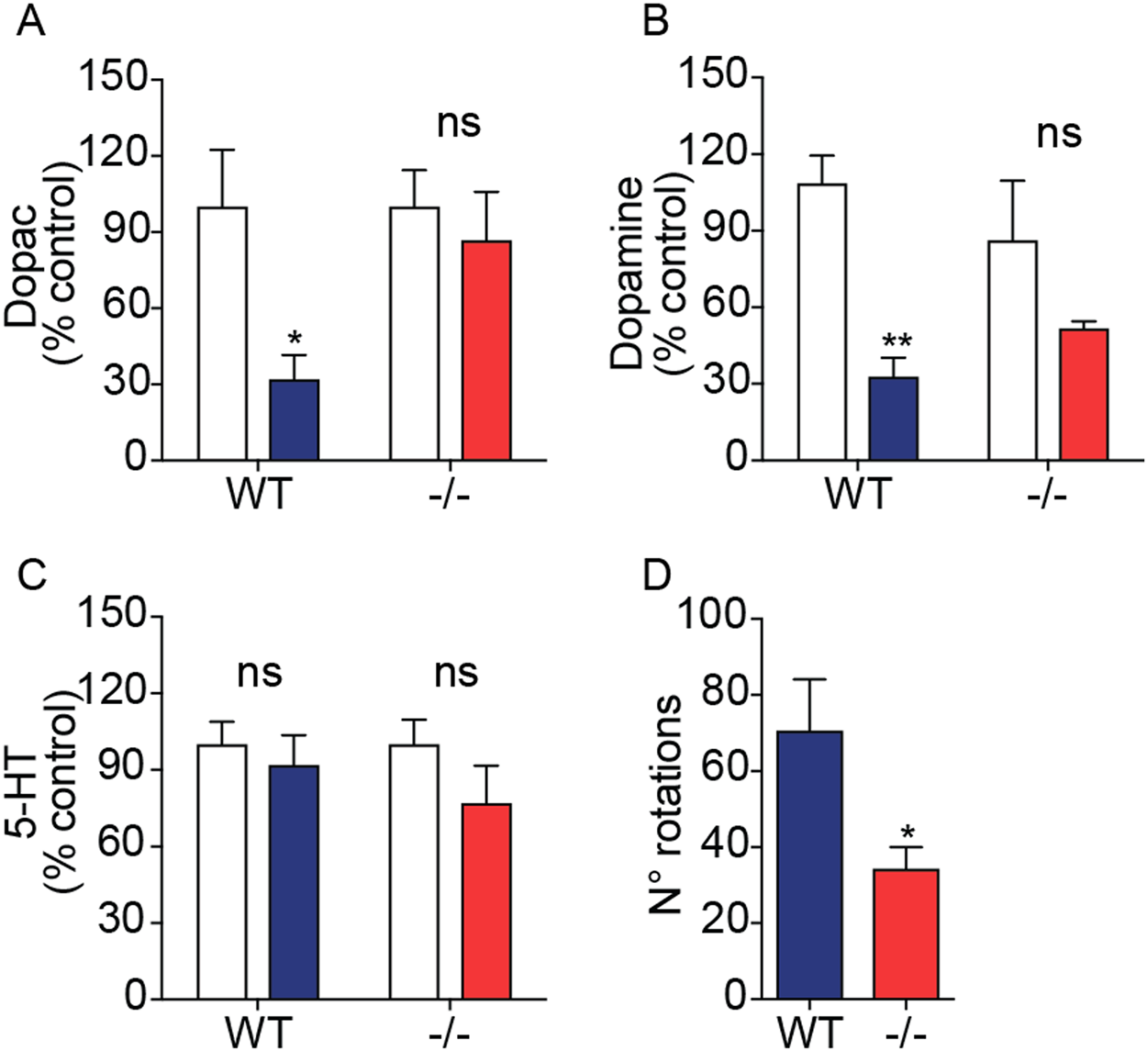
Effects of NAPE-PLD deletion on changes in striatal dopamine levels and apomorphine-induced rotations. Striatal levels of (**A**) DOPAC, (**B**) dopamine and (**C**) serotonin (5-HT) expressed as percent of intact side (n = 5); Open bars: control side of both WT and NAPE-PLD^−/−^ mice; color-coded bars: lesioned side of WT (blue) or NAPE-PLD^−/−^ (red) mice. *P < 0.05, **P < 0.01 compared to intact contralateral side, two-way ANOVA, Bonferroni post hoc test compared to intact side. (**D**) Apomorphine-induced contralateral rotations (n = 8–9); *P < 0.05 Student’s *t*-test.

Three weeks after 6-OHDA injections, when the effects of the toxin are fully expressed^36^, we immunostained TH^+^ dopamine neurons in the SN pars compacta of wild-type and NAPE-PLD^−/−^ mice. NAPE-PLD deletion appeared to be associated with greater survival of dopamine neurons (Fig. 2A,B). Supporting this conclusion, stereological analyses showed that NAPE-PLD^−/−^ mice treated with 6-OHDA lost only ≈20% of nigral dopamine neurons, compared to the ≈70% loss observed in wild-type mice (Fig. 2C). Furthermore, immunofluorescence staining of the dorsal striatum revealed that 6-OHDA injections produced a significantly greater loss of dopaminergic fibers in wild-type mice than they did in NAPE-PLD^−/−^ animals (Fig. 3). Neurochemical analyses further showed that striatal content of the dopamine metabolite 3,4-dihydroxyphenylacetic acid (DOPAC) was significantly elevated in NAPE-PLD^−/−^ mice (Fig. 4A). Dopamine levels were also partially corrected by NAPE-PLD deletion (Fig. 4B) whereas, as expected, the levels of 5-HT remained unaltered (Fig. 4C). Finally, systemic administration of the dopamine receptor agonist apomorphine (0.5 mg/kg, intraperitoneal) caused a distinctive contralateral rotation behavior in wild-type mice treated with 6-OHDA, which was attenuated in NAPE-PLD^−/−^ mice (Fig. 4D). These findings suggest that NAPE-PLD ablation increases brain NAPE content and protects from the cellular, neurochemical and behavioral consequences of 6-OHDA-induced toxicity.

### Effects of 6-OHDA on NAPE levels in human SH-SY5Y cells

**Figure 5:**
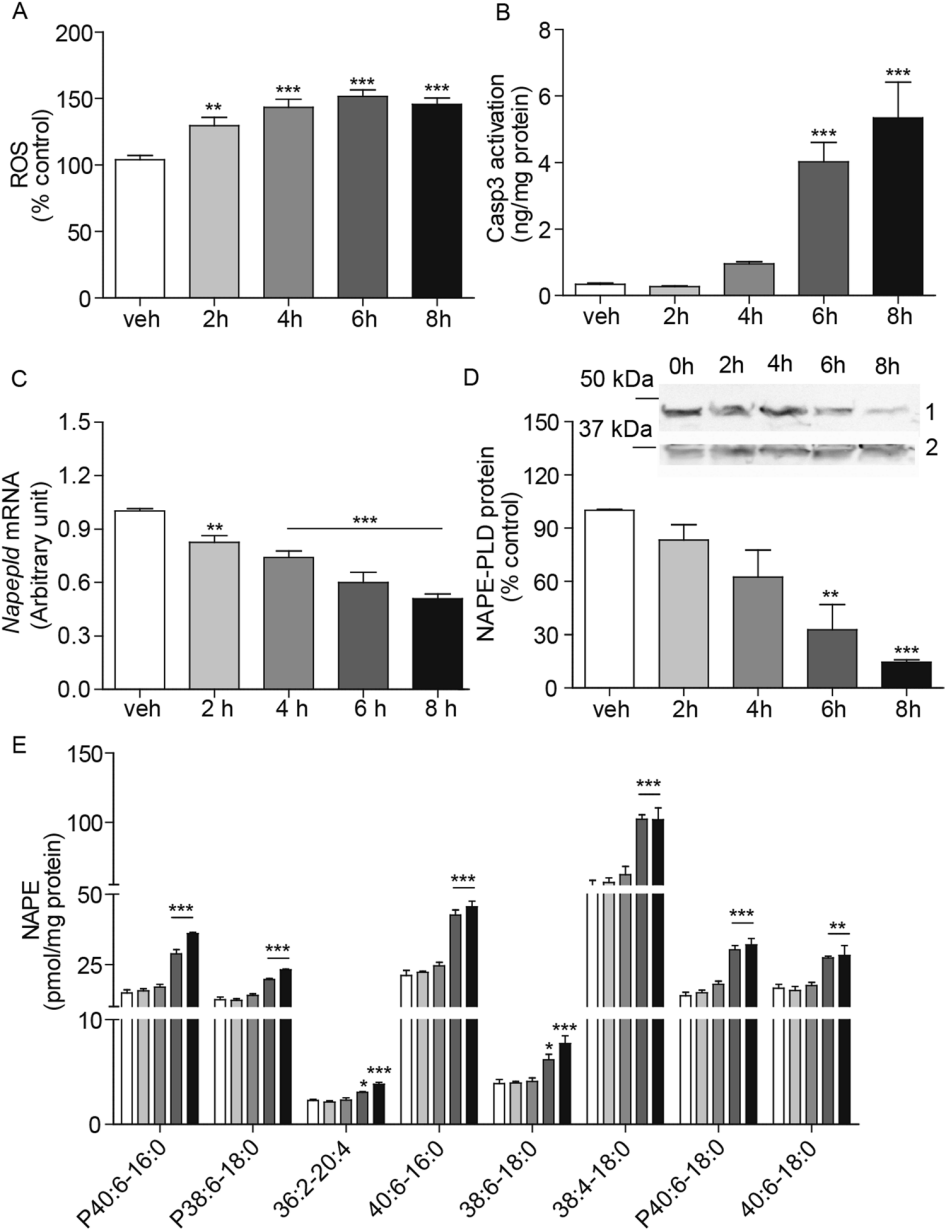
6-OHDA-induced toxicity in SH-SY5Y cells. Time-course measurements of SH-SY5Y cells incubated in the presence of 6-OHDA (100 µM) for 2 to 8 h. (**A**) Reactive oxygen species (ROS), expressed as percent of levels in vehicle-treated cells; (**B**) Active Caspase-3 (Casp3), expressed as ng per mg of protein (n = 9–14); (**C**) *Napepld* transcription, expressed as arbitrary units after normalization (see Methods) (n = 9); and (**D**) NAPE-PLD protein levels; top, representative western blot, bottom, densitometric quantification, expressed as percent of control. GAPDH was used for normalization (n = 3). Full-length blots are presented in Supplementary. (**E**) Individual NAPE levels (n = 3). *P < 0.05, **P < 0.01, ***P < 0.001, one-way ANOVA with Bonferroni post hoc test.

The human neuroblastoma cell line SH-SY5Y produces dopamine and other catecholamines and is commonly used as an *in vitro* model of dopamine neuron degeneration^37–39^. When incubated in the presence of 6-OHDA (100 μM), SH-SY5Y cells displayed an increase in reactive oxygen species (ROS) formation (Fig. 5A), which was followed by a substantial activation of the apoptosis marker caspase 3 (Fig. 5B). These effects were accompanied by a progressive down-regulation of *Napepld* gene transcription (Fig. 5C) and NAPE-PLD protein expression (Fig. 5D). Moreover, exposure to 6-OHDA caused a time-dependent increase in cellular NAPE content, which exclusively involved *N*-acyl saturated NAPE species and reached a maximum after 8 h incubation with the toxin (Fig. 5E).

### NAPE-PLD silencing increases NAPE levels in SH-SY5Y cells

**Figure 6:**
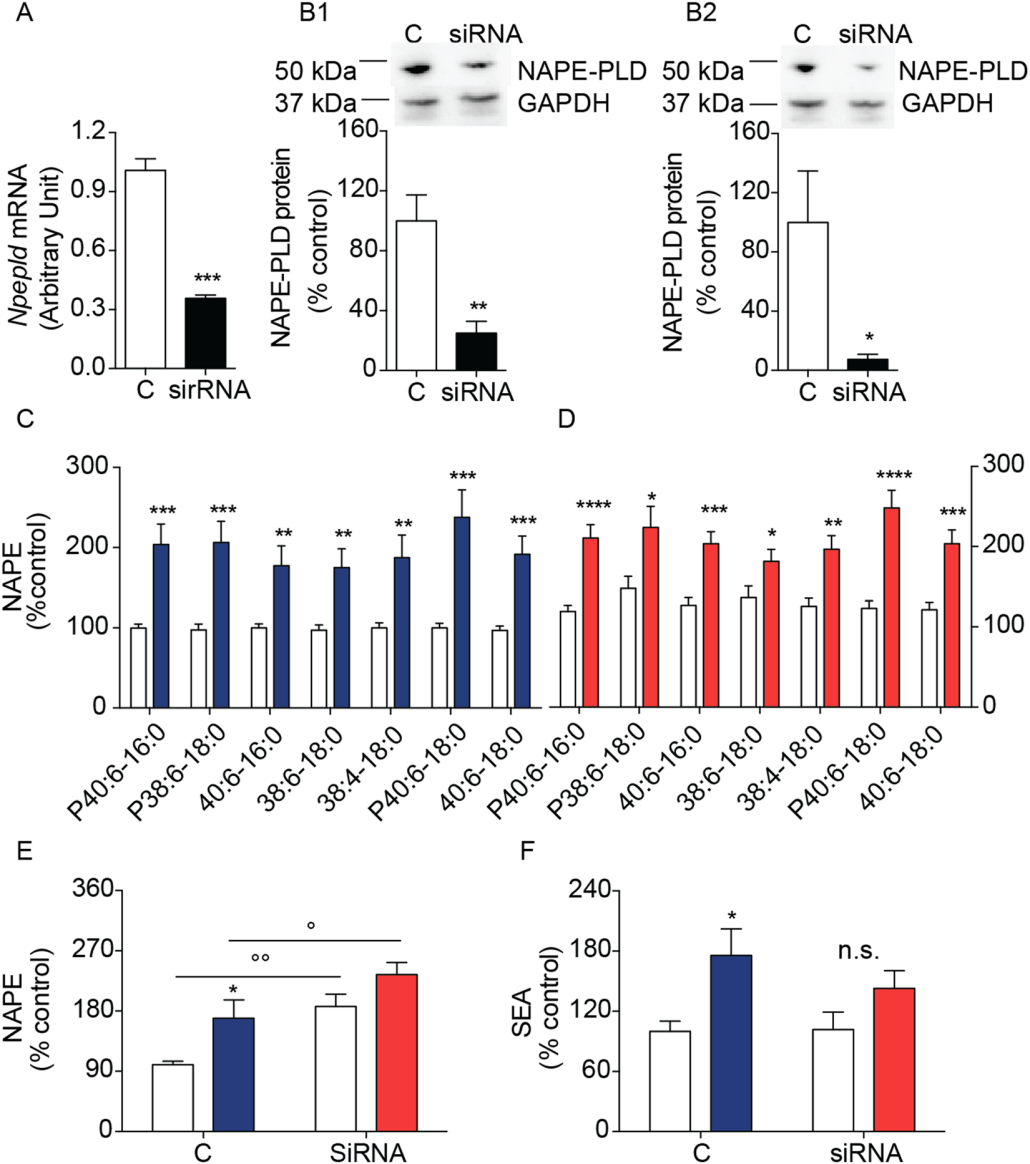
Effects of siRNA-induced NAPE-PLD silencing on NAPE and FAE levels in SH-SY5Y cells. (**A**) *Napepld* transcription and (**B1**, **B2**) NAPE-PLD protein levels in SH-SY5Y cells treated for 24 h with scrambled (C, open bar) or siRNA oligonucleotide (closed bar) (n = 8); (**B1**) membrane and (**B2**) cytosolic fractions: top, representative blot; bottom, densitometric quantification (expressed as percent control) (n = 3–5). Full-length blots/gels are presented in Supplementary. *P < 0.05, **P < 0.01, ***P < 0.001 two-tailed Student’s *t*-test. (**C**,**D**) NAPE levels in scrambled (**C**) and NAPE-PLD silenced (**D**) SH-SY5Y cells after 8 h of 6-OHDA incubation (n = 15 and 10 respectively). Open bars: control cells; color-coded bars: 6-OHDA-treated scrambled cells (blue), 6-OHDA-treated NAPE-PLD-silenced cells (red). (**E**) Total NAPE levels in SH-SY5Y cells treated for 24 h with scrambled (C) or siRNA oligonucleotide (siRNA). (**F**) SEA levels in SH-SY5Y cells treated for 24 h with scrambled (C) or siRNA oligonucleotide (siRNA). Open bars: control cells; color-coded bars: 6-OHDA-treated scrambled cells (blue), 6-OHDA-treated NAPE-PLD-silenced cells (red). *P < 0.05, **P < 0.01, ***P < 0.001 two-way ANOVA compared to scramble or vehicle-treated cells; °p < 0.05 °°p < 0.01 two-way ANOVA, Bonferroni post hoc test.

Next, we silenced the *Napepld* gene in SH-SY5Y cells using a selective 27-mer siRNA duplex, which decreased *Napepld* transcription by ≈60% compared to control cells exposed to a scrambled oligonucleotide (Fig. 6A). The observed reduction in *Napepld* mRNA was accompanied by a ≥75% decrease in the levels of NAPE-PLD protein in both cytosolic and membrane fractions (Fig. 6B)^8^, and was associated with an ≈80% increase in the levels of *N*-acyl saturated NAPE species (Fig. 6C–E). Incubation with 6-OHDA further enhanced NAPE levels in both control and siRNA-treated cells (Fig. 6C–E), but did not significantly affect SEA levels (Fig. 6F).

### NAPE-PLD silencing protects SH-SY5Y cells from 6-OHDA toxicity

**Figure 7:**
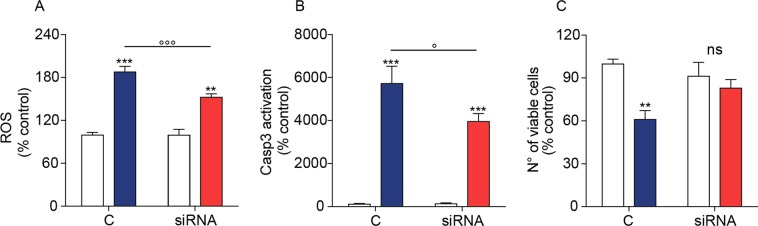
Effects of siRNA-induced NAPE-PLD silencing on 6-OHDA induced neurotoxicity in SH-SY5Y cells. (**A**) Reactive oxygen species (ROS) and (**B**) active Caspase-3 (Casp3) in control and NAPE-PLD-silenced cells measured 8 and 6 h after 6-OHDA administration (n = 8 and 4 respectively). (**C**) Number of viable cells after 8 h of incubation with 6-OHDA. Results are expressed as percent of control cells treated with vehicle (n = 3). **P < 0.01, ***P < 0.001 compared to control; °P < 0.05, °°°P < 0.001 compared to scrambled cells treated with 6-OHDA, two-way ANOVA, Bonferroni post hoc test.

To probe the functional consequences of NAPE-PLD deletion in SH-SY5Y cells, we examined the effects of siRNA-mediated NAPE-PLD silencing on 6-OHDA-induced damage. Consistent with the neuroprotective phenotype observed in NAPE-PLD^−/−^ mice, NAPE-PLD silencing significantly blunted the effects of 6-OHDA on cellular ROS production (Fig. 7A) and caspase 3 activation (Fig. 7B). Furthermore, overall cellular viability was greater in NAPE-PLD-silenced cells than in scrambled-treated controls (Fig. 7C). We interpret the results as indicating that NAPE-PLD down-regulation elevates NAPE levels and protects SH-SY5Y cells from 6-OHDA-induced toxicity.

### Mechanistic studies

**Figure 8:**
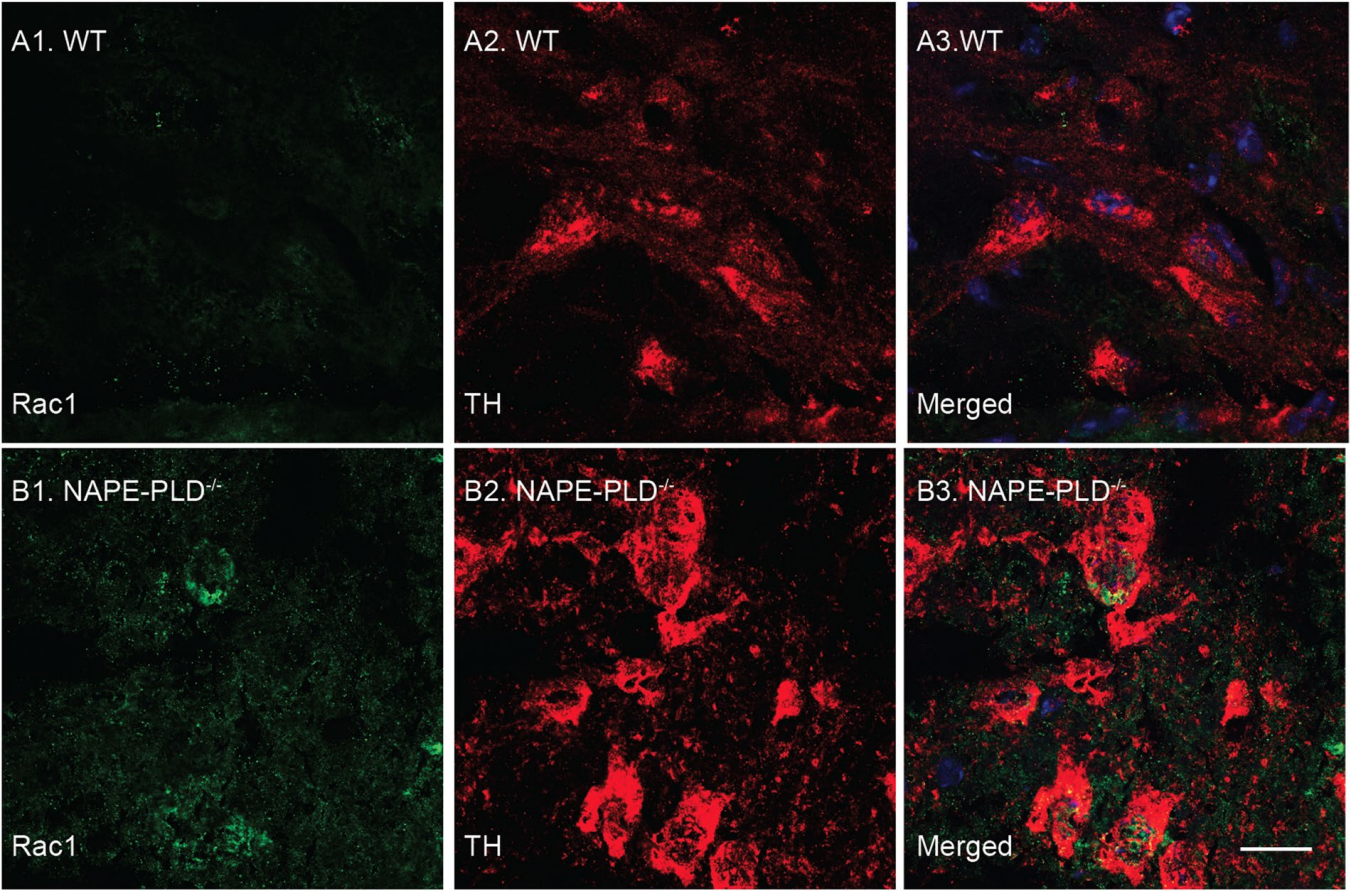
Rac1 is expressed in TH^+^ neurons of mouse SN. Immunofluorescence images for Rac1 (**A1**, **B1** green) and TH (**A2**, **B2** red) in tissue sections of SN pars compacta of (**A**) wild-type and (**B**) NAPE-PLD^−/−^ mice. Nuclei are stained with DAPI (blue). Scale bar: 20 µm.

**Figure 9:**
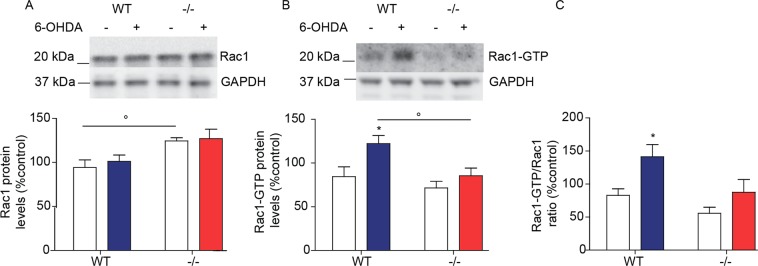
Effects of NAPE-PLD deletion on Rac1 expression and activity in mouse midbrain. Levels of (**A**) Total Rac1 protein, (**B**) GTP-bound Rac1, and (**C**) GTP-bound Rac1/total Rac1 ratio in mouse midbrain fragments containing the SN. Samples were collected 48 h after 6-OHDA. Top, representative blot; bottom, densitometric quantification. Open bars: control side; color-coded bars: lesioned side of WT (blue) or NAPE-PLD^−/−^ (red) mice (n = 5). Full-length blots/gels are presented in Supplementary. *P < 0.05, two-tailed Student’s *t*-test, °p < 0.05 two-way ANOVA, Bonferroni post hoc test.

The molecular targets of NAPEs in neural cells are unknown, but studies in mouse peritoneal macrophages have shown that treatment with exogenous NAPE (36:2–16:0) inhibits the activity of Rac1^30^, a small G protein that has been implicated in dopamine neuron survival^31,32,40^. We asked therefore whether NAPE-PLD deletion might alter Rac1 expression and function in TH^+^ dopamine neurons of the SN. Confirming previous data^32^, immunofluorescence studies showed that Rac1 is detectable in these cells (Fig. 8A). Of note, immunoreactive Rac1 levels appeared to be higher in NAPE-PLD^−/−^ mice than in wild-type controls (Fig. 8B). Confirming this finding, western blot analyses showed that total Rac1 protein content was significantly, albeit modestly, elevated in midbrain extracts of NAPE-PLD^−/−^ mice (Fig. 9A). More importantly, treatment with 6-OHDA produced an increase in the activated GTP-bound form of Rac1 in wild-type mice, but not in animals lacking NAPE-PLD (Fig. 9B,C), suggesting that accumulation of endogenous NAPEs may inhibit Rac1 activity in midbrain neurons.

Finally, to gain further insights into the molecular mechanism of action of NAPEs, we conducted a gene-expression analysis in midbrain extracts from wild-type and NAPE-PLD^−/−^ mice using an array of 84 PD-related genes (see supplementary methods for detailed list). NAPE-PLD deletion was accompanied by broad changes in gene transcription (Table S1). Interestingly, only six of the targeted genes were differentially expressed between wild-type and NAPE-PLD^−/−^ mice following 6-OHDA injection (Table 1). These include *Cadps* (Calcium Dependent Secretion Activator), *Casp9* (Caspase 9), *Egln1* (Egl-9 Family Hypoxia Inducible Factor 1), *Kcnj6* (G Protein-Activated Inward Rectifier Potassium Channel 2), *Spen* (Spen Family Transcriptional Repressor), and *Uchl1* (Ubiquitin C-Terminal Hydrolase L1). In all cases, transcription was significantly attenuated in 6-OHDA-treated NAPE-PLD^−/−^ mice, compared to 6-OHDA-treated wild-type mice (Table 1).

**Table 1:** Changes in PD-related genes transcription in wild-type (WT) and NAPE-PLD^−/−^ mice 48 h after 6-OHDA administration. Statistically detectable changes are highlighted in bold. Data are expressed as fold change (NAPE-PLD^−/−^/WT). P value was calculated using the Student’s *t* test, n = 3.

Gene Symbol	Fold regulation NAPE-PLD^−/−^/WT 6-OHDA	P value
*Aldh1a1*	−2.33	0.15
*Apc*	−1.03	0.75
*App*	−1.16	0.13
*Atp2b2*	−1.17	0.16
*Atxn2*	−1.26	0.22
*Atxn3*	−1.17	0.19
*Basp1*	−1.18	0.35
*Bdnf*	−1.61	0.11
***Cadps***	**−1**.**35**	**0**.031*
*Casp1*	−1.06	0.68
*Casp3*	−1.49	0.19
*Casp7*	−1.14	0.49
*Casp8*	−1.01	0.89
***Casp9***	**−1**.**15**	**0**.016*
*Cdc27*	−1.17	0.15
*Cdc42*	−1.08	0.51
*Cdh8*	−1.25	0.27
*Chgb*	−1.23	0.18
*Cul2*	−1.19	0.16
*Cxxc1*	−1.16	0.15
*Ddc*	−2.60	0.19
*Dlk1*	−1.66	0.07
*Drd2*	−1.79	0.17
***Egln1***	**−1**.**20**	**0**.049*
*Fbxo9*	−1.22	0.12
*Fgf13*	−1.46	0.051
*Fn1*	−1.31	0.13
*Gabbr2*	−1.10	0.64
*Gbe1*	−1.19	0.15
*Gpr37*	−1.00	0.92
*Gria3*	−1.37	0.10
*Hspa4*	−1.17	0.19
*Htr2a*	−1.49	0.08
***Kcnj6***	**−1**.**46**	**0**.025*
*Lrrk2*	−1.26	0.22
*Mapk9*	−1.17	0.22
*Mapt*	−1.13	0.19
*Ncoa1*	−1.11	0.07
*Nefl*	−1.27	0.19
*Nfasc*	−1.05	0.62
*Nr4a2*	−2.61	0.25
*Nrxn3*	−1.37	0.06
*Nsf*	−1.29	0.05
*Nsg1*	−1.25	0.20
*Ntrk2*	−1.31	0.12
*Opa1*	−1.26	0.06
*Pan2*	−1.08	0.36
*Park2*	−1.13	0.52
*Park7*	−1.25	0.18
*Pink1*	−1.25	0.11
*Ppid*	−1.15	0.33
*Prdx2*	−1.14	0.33
*Psen2*	−1.04	0.73
*Pten*	−1.25	0.08
*Rgs4*	−1.17	0.12
*Rtn1*	−1.23	0.30
*S100b*	−1.08	0.84
*Sept5*	−1.22	0.22
*Skp1a*	−1.20	0.20
*Slc18a2*	−4.27	0.16
*Slc25a4*	−1.14	0.30
*Slc6a3*	−8.32	0.20
*Slit1*	−1.18	0.57
*Snca*	−1.34	0.09
***Spen***	**−1**.**14**	**0**.041*
*Srsf7*	−1.17	0.20
*Stub1*	−1.16	0.33
*Sv2b*	−1.36	0.12
*Syngr3*	−1.25	0.22
*Syt1*	−1.38	0.21
*Syt11*	−1.18	0.09
*Tcf7l2*	−1.26	0.42
*Th*	−4.51	0.20
*Tpbg*	−1.85	0.17
*Uba1*	−1.16	0.33
*Ubc*	−1.09	0.40
*Ube2i*	−1.11	0.40
*Ube2k*	−1.21	0.34
*Ube2l3*	−1.04	0.70
***Uchl1***	**−1**.**24**	**0**.031*
*Usp34*	−1.31	0.15
*Vamp1*	−1.09	0.82
*Vdac3*	−1.13	0.26
*Ywhaz*	−1.23	0.20

## Discussion

Ischemic and toxic insults elevate NAPE levels in rodent brain tissue and neural cell cultures^23,29^, but the functional significance of this response, if any, remains unknown. In this study, we report on a possible contribution of NAPE-PLD, a zinc hydrolase that converts membrane NAPEs into FAEs^7,8^, to the neurotoxic response elicited by 6-OHDA. In mice treated with the toxin, NAPE-PLD deletion protected both dopaminergic neurons in the SN and dopamine fibers in the striatum, while attenuating the motor response to apomorphine. Furthermore, in SH-SY5Y cells incubated with 6-OHDA, NAPE-PLD silencing reduced ROS formation and caspase-3 activation, and enhanced cell viability. In both models, NAPE-PLD down-regulation was accompanied by significant increases in the levels of NAPE species containing saturated *N*-acyl substituents, but only by minor non-significant changes in FAE content. Together, the findings point to a previously unrecognized role for NAPE-PLD in the control of dopamine neuron survival, which might be mediated through regulation of membrane NAPE levels.

We have previously shown^29^, and confirmed in the present study, that unilateral injections of 6-OHDA into the mouse striatum cause a marked local accumulation of *N*-acyl saturated NAPE species such as NAPE (38:4–18:0) and NAPE (40:6–16:0). This result is consistent with published reports indicating that ischemia and other neurotoxic insults stimulate NAPE accumulation both *in vitro* and *in vivo*^41–43^. The molecular mechanism(s) underlying damage-induced NAPE accrual is unclear, but two lines of evidence suggest that changes in NAPE-PLD activity and/or expression might be involved. First, pharmacological or genetic blockade of NAPE-PLD causes profound elevations in cellular NAPE levels (^33^and present study). Second, a broad range of pro-inflammatory and tissue-damaging interventions^44–47^ – including administration of LPS^45^ or incubation of SH-SY5Y cells with 6-OHDA (present study) – suppress transcription of the *Napepld* gene, possibly by epigenetic processes similar to those recruited in macrophages^45^. Moreover, induction of focal cerebral ischemia^48^ in mouse brain is accompanied by reduced NAPE-PLD activity, suggesting that expression of this protein may be also downregulated following 6-OHDA administration. These results suggest that NAPE-PLD activity is an important contributor to NAPE homeostasis in membranes and that transcriptional suppression of *Napepld* mediates, at least in part, damage-induced NAPE accrual. Another potential mechanism may be accelerated NAPE biosynthesis, which occurs via enzyme-mediated transfer of an acyl group from the *sn*-1 position of PC to the amino group of PE^2^. This reaction is catalyzed by the cytosolic phospholipase PLA2A4E, whose activity is stimulated by calcium^3^. Since neural cell damage is almost invariably accompanied by profound alterations in intracellular calcium homeostasis, it is possible that PLA2A4E activity might also contribute to damage-induced NAPE accumulation. Future experiments will need to address this possibility.

Because NAPE-PLD catalyzes the final step in FAE biosynthesis, tissue levels of these lipid molecules are often, but not always, lower in mice lacking NAPE-PLD than they are in wild-type mice^33^. In the present study, we did not observe any significant difference in striatal FAE levels between NAPE-PLD^−/−^ and wild-type mice or between NAPE-PLD-silenced SH-SY5Y cells and their controls. Similarly, pharmacological inhibition of NAPE-PLD activity in intact Hek293 cells was found to cause a substantial accumulation of NAPEs without changes in FAE content^49^. These results are consistent with the non-rate limiting role of NAPE-PLD in FAE biosynthesis^8^, which is most likely controlled by PLA2A4E activity^1,50^. However, the available data do not allow us to exclude that compensations may occur in mice lacking NAPE-PLD, which might offset the genetic removal of this enzyme via isofunctional substitution. The existence of such compensations has been documented, for example, in bone marrow macrophages isolated from NAPE-PLD^−/−^ mice^45^. Despite these uncertainties, our results clearly show that NAPE-PLD deletion increases the levels of *N*-acyl saturated NAPE species (e.g., 38:4–18:0) without affecting those of the corresponding FAEs (e.g., SEA). As such, the findings raise the intriguing possibility that NAPE-PLD activity might regulate dopamine neuron survival through its ability to control NAPE homeostasis in membranes. Testing this hypothesis will require further experimentation, but biophysical studies with synthetic NAPEs – such as 1,2-dioleoyl-phosphatidylethanolamine *N*-dodecanoyl [NAPE (36:2–12:0)] and 1,2-myristoyl-phosphatidylethanolamine *N*-myristoyl [NAPE (28:0–14:0)] – in reconstituted systems suggest several possible ways by which NAPEs might affect cell function, which include stimulation of calcium-dependent membrane fusion^19^, consolidation of lipid raft structure^20^ and stabilization of the lipid bilayer^17,18^. Akin to the phosphoinositides^21^, the NAPEs might also regulate the association of cytosolic proteins to the internal facet of the cell membrane^22^.

An initial exploration of the molecular mechanisms underlying the neuroprotective effects of NAPEs suggests that multiple factors are likely to be involved. Previous work has shown that treatment with exogenous NAPE (36:2–16:0) inhibits the activity of Rac1 in peritoneal mouse macrophages and J774A.1 cells^30^. Rac1 is a Rho family small G protein that has been implicated, among other functions, in dopamine neuron survival and Parkinson’s disease^32,40^. These data prompted us to ask whether NAPE-PLD deletion and consequent membrane NAPE accrual might influence Rac1 activity in mouse brain. We confirmed that midbrain dopamine neurons express Rac1, and further found that intrastriatal 6-OHDA injections cause an increase in the amount of activated GTP-bound form of Rac1 in the SN of wild-type mice, but not of mice lacking NAPE-PLD. This result points to a possible role for Rac1 in 6-OHDA-induced neurotoxicity, and suggests that deactivation of this small G protein may contribute to the neuroprotective effects of NAPE-PLD deletion. The mechanism through which NAPE-PLD deletion regulates Rac1 activity is unknown, though one possibility is that accrual of membrane NAPE levels might influence the association of Rac1 to cell membranes, which is required for GDP to GTP exchange and Rac1 activation^51^.

It seems unlikely that Rac1 regulation is the only mechanism through which NAPEs influence neuronal viability. Indeed, the focused gene array study presented in Table 1 identified six genes whose transcription may be significantly attenuated by 6-OHDA in midbrain extracts of NAPE-PLD^−/−^ mice compared to wild-type controls. These include *Cadps* (Calcium Dependent Secretion Activator), a peripheral membrane protein involved in vesicle fusion and monoamine neurotransmission^52^ and Parkinson’s disease pathogenesis^53^; *Casp9* (Caspase 9) a mediator of apoptosis whose activity may be elevated in peripheral blood cells of persons with sporadic Parkinson’s disease^54^; *Egln1* (Egl-9 Family Hypoxia Inducible Factor 1), whose transcription may be enhanced in the SN of sporadic PD patients^55,56^; *Kcnj6* (G Protein-Activated Inward Rectifier Potassium Channel 2), whose transcription may also be enhanced in the SN of sporadic Parkinson’s disease patients^57^; *Spen* (Spen Family Transcriptional Repressor) a hormone inducible repressor; and, finally, *Uchl1* (Ubiquitin C-Terminal Hydrolase L1), a key component of the ubiquitin-proteasome pathway that is mutated in some familial forms of Parkinson’s disease^58^. The multiplicity of molecular effectors associated with NAPE-PLD deletion makes it difficult to identify a univocal mechanism through which membrane NAPEs might influence cell death-related signals (e.g., ROS, caspase 3 activation) and dopamine neuron survival. Such multiplicity might reflect a pleiotropic role for membrane NAPEs in intracellular signaling, which is further supported by the broad effects exerted by these lipid molecules on membrane structure and function (discussed above).

Another question raised by the present results pertains to the functional significance of NAPE heterogeneity, which involves substituents in the *sn-1* (16:0/18:0), *sn-2* (20:4/22:6) and *N*-position (16:0/18:0). Such heterogeneity is well established in the literature (see for example^23,28,59^), but can be only partially attributed to species and tissue variability. Other factors are likely to play a role, including the potential role of NAPEs as either FAE precursors or autonomous membrane signals. Additional work is needed to address these intriguing possibilities.

Abnormalities in lipid composition have emerged as important pathogenic factors in neurodegenerative disorders such as Parkinson’s disease^60^ and Alzheimer’s disease^61^. For example, mutations in the glycolipid-metabolizing enzyme glucocerebrosidase (*GBA1*) are common in the familiar forms of Parkinson’s disease, while single-nucleotide polymorphisms of genes involved in other aspects of lipid metabolism [e.g., *ASAH1*^62^, *PLA2G6*^63^ and *SMPD1*^64^] have been detected in the sporadic and more frequent form of this disorder. Furthermore, genome-wide association studies have identified several lipid-processing genes (e.g., *APOE4*, *PLD3* and *ABCA7*) as risk loci that may increase susceptibility for Alzheimer’s disease^61^. Indeed, select alterations in plasma phospholipid profile were shown to predict, with over 90% accuracy, phenoconversion of cognitively normal older adults to either Alzheimer’s disease or mild cognitive impairment^65^. In this context, it is worth noting that that the activities of two enzymes – phosphoethanolamine cytidylyltransferase and phosphocholine cytidylyltransferase – that are rate-limiting for the biosynthesis for the NAPE precursors, PC and PE, are abnormally elevated in the SN of persons with Parkinson’s disease^66^. By revealing a protective role for NAPE-PLD in neurodegeneration, the present results underscore the need for further and more detailed explorations in the lipidomics of neurodegenerative disorders.

In sum, we have shown that complete or partial genetic deletion of NAPE-PLD, the membrane-associated zinc hydrolase that converts NAPEs into FAEs, increases NAPE levels and concurrently protects mouse dopamine neurons and human dopamine-producing SH-SY5Y cells from the neurotoxic effects of 6-OHDA. The results suggest that NAPE-PLD activity may participate in the regulation of dopamine neuron survival, possibly by controlling membrane NAPE homeostasis.

## Materials and Methods

### Animals

All procedures were performed in accordance with the Ethical Guidelines of the European Union (directive 2010/63/EU of 22 September 2010) and were approved by Italian Ministry of Health (DDL 26/2014 and previous legislation; protocol number 095/2015). NAPE-PLD^−/−^ mice were generated on a C57BL6J background as previously described^33^. All mice were group-housed in ventilated cages and had free access to food and water. They were maintained under a 12 h light/dark cycle (lights on at 8:00 am) at controlled temperature (21 ± 1 °C) and relative humidity (55% ± 10%). All efforts were made to minimize animal suffering and to use the minimal number of animals required to produce reliable results.

### Chemicals

6-OHDA hydrochloride, chloral hydrate, ketamine, xylazine, paraformaldehyde (PAF), dopamine, serotonin (5-HT), DOPAC and ascorbic acid were purchased from Sigma Aldrich (Milan, Italy). NAPE standards and internal standards were synthetized in the laboratory as previously described^29^.

### Cell cultures

SH-SY5Y cells were obtained from Sigma Aldrich and were cultured at 37 °C and 5% CO_2_ in Dulbecco’s Modified Eagle’s Medium (DMEM) (Euroclone, Milan, Italy) supplemented with 10% fetal bovine serum (FBS, Thermo Fisher, Waltham, MA, USA), L-glutamine (2 mM) and antibiotics (Euroclone). Cells were treated with 6-OHDA (100 μM), or vehicle (saline containing 0.2% ascorbic acid) for the indicated times.

### NAPE-PLD silencing

siRNA experiments were performed using a *Napepld*-specific 27mer siRNA duplex (Origene, Rockville, MD, USA). A siRNA duplex carrying TYE-563 fluorescence was used to monitor transfection. A siRNA duplex carrying a 27-mer sequence targeting the hypoxanthine phosphoribosyltransferase 1 (HPRT) gene was used as positive control. Scrambled siRNA was included in each experiment as negative control. *Napepld* siRNA complexes (10 nM) were formed by mixing siRNA with lipofectamine (Invitrogen, Carlsbad, CA, USA) for 10 min at room temperature and then added to SH-SY5Y cells cultured with 1% FBS Optimem medium (Gibco, Waltham, MA, USA). Cells were incubated with siRNA oligonucleotide for 6 h. After an 18 h incubation with fresh full-growth medium, 6-OHDA (100 μM) was added for additional 8 hours.

### Surgical procedures

Mice were anesthetized with a mixture of ketamine (87.5 mg-kg^−1^) and xylazine (12.5 mg-kg^−1^; 0.1 ml per 20 g body weight, intraperitoneal, i.p.) and placed in a stereotaxic frame with a mouse adaptor (Kopf Instruments, Tujunga, CA, USA). 6-OHDA was dissolved at a fixed concentration of free base (3.2 mg-ml^−1^) in ice-cold saline containing ascorbic acid (0.02% weight/volume). Two 6-OHDA injections (1 μl each) were made using a 33-gauge Hamilton syringe (Hamilton, Reno, NV, USA) at the following brain coordinates: (*i*) AP = +1.0, L = −2.1, DV = −2.9; and (*ii*) AP = +0.3, L = −2.3, DV = −2.9^67^. Injections were performed at a rate of 0.25 μl-min^−1^ and 2 min were allowed for the toxin to diffuse.

### Lipid extraction and LC/MS analyses

NAPE and FAE levels in cells and tissues were measured as previously described^29^ (for further details see Supplementary).

### Tissue processing and immunohistochemistry

Mice were deeply anaesthetized with chloral hydrate (450 mg-kg^−1^, i.p.) and perfused transcardially with ice-cold sterile saline (20 ml), followed by ice-cold PFA [4% in phosphate-buffered saline (PBS), 60 ml]. The brains were excised and stored in a sucrose solution (25% in PBS) at 4 °C. Three series of sections (thickness: 40 µm) were collected in the coronal plane using a cryostat, and stored at −20 °C. Single immunostaining protocols were performed by incubation with primary antibody (for details see Table S3) followed by secondary Alexa Fluor 546 or 488 antibodies (1:1000; Invitrogen Carlsbad, CA, USA). Multiple labeling were conducted sequentially. Images were collected using a Nikon A1 confocal microscope with a 10 1.4 numerical aperture objective lens. Quantification of TH optical density was performed using the ImageJ software.

### Stereological measurements

Dopamine neurons of the SN were identified after TH staining followed by Alexa fluor 488 secondary antibody of midbrain regions with a 4x objective. The SN was outlined using the Paxinos and Franklin’s mouse brain atlas as reference^67^. TH^+^ neurons were counted in every 6th section. Briefly, unbiased sampling and blinded stereological counting were performed using the optical fractionator probe of the Stereo Investigator software (MBF Bioscience, Williston, VT, USA). Parameters used included a 60x oil objective, a counting frame size of 60 × 60, a sampling site of 100 × 100, a dissector height of 15 μm, 2 μm guard zones. The Gunder’s coefficient of error was less than 0.1. A total of 4 animals per group were used and 5 to 8 sections per animal were counted in the red channel.

### Western blot analyses

Cell pellets were homogenized in 150 μl of a radioimmunoprecipitation assay buffer (RIPA), consisting of 50 mM Tris-HCI (pH 7.4), 1% Tryton X 100, 0.5% sodium-deoxycholate, 0.1% sodium dodecyl sulfate, 150 mM sodium chloride, 2 mM ethylenediaminetetraacetic acid. Protein concentrations were measured using the bicinchoninic acid (BCA) method, following manufacturer’s instructions (Thermo Fisher Scientific). Proteins (30 µg) were denatured in SDS (8%) and β-mercaptoethanol (5%) at 95 °C for 5 min. After separation by SDS-PAGE on a 4–15% gel, the proteins were electrotransferred to nitrocellulose membranes. The membranes were blocked with 5% non-fat dry milk in tris-buffered saline (TBS) and incubated overnight with primary antibodies (for details see Table S3) in 1% milk-TBS containing 0.1% Tween-20, followed by incubation with horseradish peroxidase-linked to the secondary antibody (1:5,000, Millipore) in TBS 0.1% Tween-20 at room temperature for 1 h. Finally, proteins were visualized using an ECL kit (Bio-Rad, USA) and the chemiluminescence image was recorded using a LAS-4000 lumino-image analyzer system (Fujifilm, Tokyo, Japan).

### Neurotransmitter measurements

Striatal tissue was removed, snap frozen in liquid N_2_ and stored at −80 °C. Samples were weighed and homogenized in 0.9 ml/methanol:water (1:1) containing 0.1% formic acid. After stirring and centrifugation, the supernatants were dried under N_2_ and the samples were reconstituted in 90 µl of mobile phase A (0.1% acetic acid in water) for LC-MS/MS analyses. LC-MS/MS analyses were carried out on an Acquity UPLC system coupled with a Xevo TQ-MS triple quadrupole mass spectrometer. Chromatographic separation was achieved using a BEH C18 column (2.1 × 100 mm, 1.7 μm particle size) eluted at a flow rate of 0.35 mL/min, using the following gradient conditions: 0–1.0 min 5% solvent B (methanol) in solvent A (0.1% acetic acid in water), 1.0–2.5 min 5% to 100% B, and 2.5–3.5 min 100% B. The column was re-equilibrated to initial conditions from 3.5 to 4.5 min. Total run time for analysis was 4.5 min, and injection volume was 5 μL. The column temperature was kept at 45 °C. The MS was operated in both positive and negative ESI mode with cone voltage, collision energy and capillary voltage set at 10 V, 20 V and 3kv respectively. The source temperature was 120 °C. Desolvation gas and cone gas (nitrogen) flow were set at 800 and 50 l/h, respectively. Desolvation temperature was 450 °C. Analytes were quantified by MRM with the following transitions (*m/z*): dopamine, 153.8 > 136.7; serotonin 176.9 > 159.9; DOPAC, 166.5 > 122.9. Dopamine and serotonin were acquired in positive mode and DOPAC in negative mode. Data were acquired by MassLynx software and quantified by TargetLynx software; individual standard calibration curves were used for the quantification of each analyte.

### Apomorphine-induced rotations

Three weeks after 6-OHDA administration groups of 8 to 9 mice received subcutaneous injections of apomorphine (0.5 mg/kg, dissolved in saline containing 0.2 mg-mL^−1^ ascorbic acid) and motor behavior was evaluated for the following 60 min. Rotational asymmetry was assessed using the ANY-maze Behavior Tracking Software (Stoelting Europe, Dublin, Ireland). Only full body turns were counted^68^. Data are presented as total number of net turns in 1 h, with rotation toward the side contralateral to the lesion given a positive value.

### Real-time quantitative PCR

Total RNA was prepared from pellets of SH-SY5Y cells (2.5 × 10^5^ cells) as previously described^69^ (for further details see Supplementary). The BestKeeper software^70^ was used to determine expression stability and geometric mean of two different housekeeping genes (HMBS and HPRT). ΔCt values were calculated by subtracting the Ct value of the geometric mean of these housekeeping genes from the Ct value for the genes of interest. The relative quantity of genes of interest was calculated by the expression 2−ΔΔCt. Results are reported as fold induction over control.

### Array mRNA measurements

Midbrain samples from the contralateral or ipsilateral side of 3 wild-type and 3 NAPE-PLD^−/−^ mice, were used for mRNA analysis. mRNA was extracted as described above, and cDNA synthesis carried out with RT^2^ First Strand Kit (Qiagen, Milan, Italy) using 0.25 µg of purified mRNA and according to the manufacturer’s instructions. First-strand cDNA was loaded on the RT^2^ Profiler PCR Array (Qiagen, Cod. 330231 PAMM-124ZA) and run at 95 °C for 10 min followed by 40 cycles, each cycle consisting of 15 sec at 95 °C and 1 min at 60 °C, using a ViiA7 instrument (ViiA™ 7 Real-Time PCR System, LifeTechnologies). Data analysis was performed with the SABiosciences PCR Array Data Analysis software (www.SABiosciences.com/pcrarraydataanalysis.php). Genes analyzed are reported in Table S4.

### Intracellular ROS measurements

Relative changes in intracellular ROS were monitored in SH-SY5Y cells using the fluorescent probe dichloro-dihydro-fluoroscein diacetate (DCFH-DA) (Abcam, Cambridge, UK). Cells were grown in 24 multi-well plates and, before treatment with 6-OHDA, were incubated with 5 μM DCFDA for 1 h at 37 °C. At the end of the treatment with 6-OHDA, cells were harvested, transferred to black multi-well plates and fluorescence was measured using a Tecan microplate reader with excitation-emission set to 485–535 nm.

### Activated caspase-3 measurements

An ELISA kit (R&D, Abingdon, UK) was used to measure levels of activated caspase-3 in SH-SY5Y cells. The assay was performed following manufacturer’s instructions.

### Cell viability assay

SH-SY5Y cells were harvested, diluted in PBS and counted using a Scepter^TM^ cell counter (Merck Millipore, Darmstadt, DE), considering cells of similar morphology and size.

### Statistical analyses

Data were analyzed using GraphPad Prism version 5 for Windows (La Jolla, California, USA). Parametric statistical analysis was performed using the two-tailed Student’s *t*-test for two groups; one-way or two-way analysis of variance (ANOVA) was applied for multiple comparisons with Bonferroni post hoc analysis for data meeting homogeneity of variance. Differences between groups were considered statistically significant at values of P < 0.05. Results are expressed as mean ± S.E.M.

## Supplementary information

Supplementary info

## Data Availability

The datasets generated during the current study are available from the corresponding author on reasonable request.
