# Supplementary material for: A protective role for N-acylphosphatidylethanolamine phospholipase D in 6-OHDA-induced neurodegeneration
Source: Sci Rep. 2019 Nov 4;9:15927. doi: 10.1038/s41598-019-51799-1 (PMC6828692; doi:10.1038/s41598-019-51799-1)

# A protective role for N-acylphosphatidylethanolamine phospholipase D in 6-OHDA-induced neurodegeneration

Francesca Palese[1,2], Silvia Pontis[1], Natalia Realini[1], Daniele Piomelli*[2]

[1] Department of Drug Discovery and Development,
Fondazione Istituto Italiano di Tecnologia, via Morego 30, 16163 Genoa, Italy

[2] Departments of Anatomy and Neurobiology, Pharmacology and Biological Chemistry,
University of California, Irvine, CA 92697-4625, USA

**SUPPLEMENTARY TABLE**

**Supplementary Table 1:** Changes in PD-related genes transcription in control wild-type (WT) and NAPE-PLD$^{-/-}$ mice. Data are expressed as fold change (WT/NAPE-PLD$^{-/-}$). P value was calculated using the Student's *t* test, n = 3.

| Gene Symbol | Fold Regulation WT/NAPE-PLD$^{-/-}$ Control | P-value |
|---|---|---|
| *Aldh1a1* | -2.78 | 0.25 |
| *Apc* | -1.75 | 0.018 * |
| *App* | -1.61 | 0.029 * |
| *Atp2b2* | -1.38 | 0.28 |
| *Atxn2* | -1.49 | 0.014 * |
| *Atxn3* | -1.68 | 0.020 * |
| *Basp1* | -1.32 | 0.12 |
| *Bdnf* | -1.65 | 0.045 * |
| *Cadps* | -1.64 | 0.025 * |
| *Casp1* | -1.31 | 0.47 |
| *Casp3* | -2.05 | 0.025 * |
| *Casp7* | -1.64 | 0.12 |
| *Casp8* | -1.23 | 0.33 |
| *Casp9* | -1.80 | 0.037 * |
| *Cdc27* | -1.33 | 0.057 |
| *Cdc42* | 1.07 | 0.52 |
| *Cdh8* | 1.23 | 0.44 |
| *Chgb* | -1.31 | 0.20 |
| *Cul2* | -1.30 | 0.06 |
| *Cxxc1* | -1.37 | 0.04 * |
| *Ddc* | -2.55 | 0.17 |
| *Dlk1* | -1.87 | 0.004 ** |
| *Drd2* | -1.86 | 0.13 |
| *Egln1* | -1.57 | 0.027 * |
| *Fbxo9* | -1.47 | 0.014 * |
| *Fgf13* | -1.76 | 0.023 * |
| *Fn1* | -2.28 | 0.051 |
| *Gabbr2* | -1.31 | 0.55 |
| *Gbe1* | -1.45 | 0.008 * |
| *Gpr37* | -1.06 | 0.95 |
| *Gria3* | -1.62 | 0.047 * |

| | | |
|---|---|---|
| *Hspa4* | -1.57 | 0.009 ** |
| *Htr2a* | -1.22 | 0.41 |
| *Kcnj6* | -1.73 | 0.005** |
| *Lrrk2* | 1.29 | 0.19 |
| *Mapk9* | -1.28 | 0.27 |
| *Mapt* | -1.53 | 0.007 ** |
| *Ncoa1* | -1.64 | 0.030 * |
| *Nefl* | -1.37 | 0.17 |
| *Nfasc* | -1.65 | 0.017 * |
| *Nr4a2* | -2.01 | 0.40 |
| *Nrxn3* | -1.63 | 0.032 * |
| *Nsf* | -1.55 | 0.09 |
| *Nsg1* | -1.57 | 0.028 * |
| *Ntrk2* | -1.70 | 0.020 * |
| *Opa1* | -1.28 | 0.63 |
| *Pan2* | -1.51 | 0.025 * |
| *Park2* | -1.92 | 0.028 * |
| *Park7* | -1.39 | 0.002 ** |
| *Pink1* | -1.72 | 0.025 * |
| *Ppid* | 1.34 | 0.052 |
| *Prdx2* | -1.32 | 0.12 |
| *Psen2* | -1.08 | 0.43 |
| *Pten* | -1.23 | 0.18 |
| *Rgs4* | -1.46 | 0.33 |
| *Rtn1* | -1.55 | 0.001 ** |
| *S100b* | -1.27 | 0.017 * |
| *Sept5* | -1.60 | 0.028 * |
| *Skp1a* | -1.54 | 0.030 * |
| *Slc18a2* | -2.27 | 0.34 |
| *Slc25a4* | -1.33 | 0.06 |
| *Slc6a3* | -9.03 | 0.29 |
| *Slit1* | -1.36 | 0.26 |
| *Snca* | -1.42 | 0.043 * |
| *Spen* | -1.40 | 0.003 ** |
| *Srsf7* | -1.63 | 0.011 * |
| *Stub1* | -1.38 | 0.057 |
| *Sv2b* | -1.67 | 0.25 |
| *Syngr3* | -1.68 | 0.012 * |
| *Syt1* | -1.58 | 0.24 |
| *Syt11* | -1.72 | 0.036 * |

| | | |
|---|---|---|
| *Tcf7l2* | 1.01 | 0.85 |
| *Th* | -2.98 | 0.23 |
| *Tpbg* | -1.99 | 0.004 ** |
| *Uba1* | -1.45 | 0.009 ** |
| *Ubc* | -1.40 | 0.06 |
| *Ube2i* | -1.51 | 0.003 ** |
| *Ube2k* | -1.38 | 0.08 |
| *Ube2l3* | -1.24 | 0.026 * |
| *Uchl1* | 1.23 | 0.13 |
| *Usp34* | -1.85 | 0.004 ** |
| *Vamp1* | 1.01 | 0.82 |
| *Vdac3* | -1.38 | 0.028 * |
| *Ywhaz* | -1.82 | 0.032* |

## SUPPLEMENTARY METHODS

**Lipid extraction and liquid chromatography/mass spectrometry (LC/MS) analysis.** Briefly, snap-frozen cell pellets or pre-weighed tissues were homogenized in a chloroform:methanol mixture (1:2 v/v, 2 ml) containing 18:0-22:6-17:0 NAPE [synthetized in the lab[29]] and deuterated stearoylethanolamide (d3-SEA) as internal standards. Lipids were extracted twice with chloroform (0.6 mL) and the organic phases were washed with water (0.6 mL), collected, and dried under $N_2$. The organic extracts from cells were reconstituted in a mix of methanol/chloroform (9:1 v/v, 0.1 mL). The organic extracts from tissues were fractionated by silica gel column chromatography[5] and NAPEs and FAEs were eluted with 2 mL chloroform/methanol (1:1, 9:1 v/v respectively). Organic phases were evaporated under $N_2$ and reconstituted in 0.1 mL of methanol/chloroform (9:1, v/v). LC/MS analyses were conducted on a Xevo TQ LC-MS/MS system equipped with a reversed-phase HSS T3 column (2.1 x 50 mm) (Waters, Milford MA), using gradient elution (1 min 95% A and 5% B followed by 7 min 100% B and again 2 min 95% A and 5% B) at a flow rate of 0.4 mL/min. The mobile phase consisted of solvent A (acetonitrile plus 10 mM ammonium formate in water, 60:40, v/v) and solvent B (acetonitrile plus 10 mM ammonium formate in isopropyl alcohol, 10:90, v/v). The capillary voltage was set at 3kV. The cone voltage was 25V. The source temperature was 120°C. Desolvation gas and cone gas ($N_2$) flow were set to 800 and 20 L/h, respectively. Desolvation temperature was 450°C. Detection and analysis were controlled by the Waters MassLynx software version 4.1. Multiple reaction monitoring (MRM) transitions for all analytes are reported in **Table S2**. As this LC/MS-MS protocol does not allow one to differentiate *sn*-1 from *sn*-2 substituents, individual NAPE species are designated below as NAPE (X:Y-N-acyl), where X is the total number of carbon atoms and Y the total number of double bonds in the *sn*-1 and *sn*-2 chains.

**Table S2:** MRM transitions and MS parameters for NAPE and FAE species. P, plasmalogen.

| NAPE | Parent Ion *(m/z)* | Daughter Ion *(m/z)* | Cone Voltage (V) | Collision Energy (V) |
|---|---|---|---|---|
| **P40:6-N16:0** | 1014.8 | 282.3 | 25 | 20 |
| **P38:6-N18:0** | 1014.8 | 310.3 | 25 | 20 |
| **40:6-N16:0** | 1030.8 | 282.3 | 25 | 20 |
| **36:2-20:4** | 1030.8 | 324 | 25 | 20 |
| **38:6-N18:0** | 1030.8 | 310.3 | 25 | 20 |
| **38:4-N18:0** | 1034.8 | 310.3 | 25 | 20 |
| **P40:6-N18:0** | 1042.8 | 310.3 | 25 | 20 |
| **40:6-N18:0** | 1058.8 | 310.3 | 25 | 20 |
| **34:1-16:0** | 920.3 | 282 | 25 | 20 |
| **34:1-18:0** | 957.2 | 310.3 | 25 | 20 |
| **40:6-N17:0** | 1044.6 | 296.4 | 25 | 20 |
| **SEA** | 328 | 62 | 20 | 30 |
| **d3-SEA** | 331 | 62 | 20 | 30 |

**Table S3:** Antibodies used

| **Antibody** | **Host specie** | **Brand** | **Code** | **Technique** | **Dilution** |
|---|---|---|---|---|---|
| Anti-TH | Chicken | Abcam | Ab76442 | IF | 1:500 |
| Anti-NAPE-PLD | Rabbit | Abcam | Ab95397 | WB | 1:200 |
| Anti- Rac1 | Mouse | Abcam | Ab33186 | WB<br>IF | 1:500<br>1:200 |
| Anti-Rac1-GTP | Mouse | New East Biosciences | 26903 | WB | 1:1000 |
| Anti-GAPDH | Rabbit | Abcam | Ab9485 | WB | 1:2000 |

**Real-time quantitative PCR.** Total RNA was prepared from pellets of SH-SY5Y cells ($2.5x10^5$ cells) using the Ambion PureLink RNA minikit (Life Technologies, Carlsbad, CA, USA) as directed by the supplier. Samples were treated with DNase (PureLink DNase, Life Technologies) and cDNA synthesis was carried out using the Super-Script VILO cDNA synthesis kit (Life Technologies) according to the manufacturer's protocol using purified RNA (0.5-1 µg). First-strand cDNA was amplified using the TaqMan 5′ nuclease activity from the TaqMan Universal PCR Master Mix, fluorogenic probes, and oligonucleotide primers. Copy numbers of cDNA targets were quantified of the point during cycling when the PCR product was first detected. Gene-specific primers for Taqman assays were purchased from Life Technologies. Quantitative PCR was performed in 96-well PCR plates and run at 95°C for 10 min, followed by 40 cycles, each cycle consisting of 15 s at 95°C and 1 min at 60°C, using a ViiA7 instrument (ViiATM 7 real-time PCR system, Life Technologies).

**Table S4:** RT2 Profiler PCR Array genes

| Position | Symbol | Description |
|---|---|---|
| A01 | *Aldh1a1* | Aldehyde dehydrogenase family 1, subfamily A1 |
| A02 | *Apc* | Adenomatosis polyposis coli |
| A03 | *App* | Amyloid beta (A4) precursor protein |
| A04 | *Atp2b2* | ATPase, Ca++ transporting, plasma membrane 2 |
| A05 | *Atxn2* | Ataxin 2 |
| A06 | *Atxn3* | Ataxin 3 |
| A07 | *Basp1* | Brain abundant, membrane attached signal protein 1 |
| A08 | *Bdnf* | Brain derived neurotrophic factor |
| A09 | *Cadps* | Ca2+-dependent secretion activator |
| A10 | *Casp1* | Caspase 1 |
| A11 | *Casp3* | Caspase 3 |
| A12 | *Casp7* | Caspase 7 |
| B01 | *Casp8* | Caspase 8 |
| B02 | *Casp9* | Caspase 9 |
| B03 | *Cdc27* | Cell division cycle 27 homolog (*S. cerevisiae*) |
| B04 | *Cdc42* | Cell division cycle 42 homolog (*S. cerevisiae*) |
| B05 | *Cdh8* | Cadherin 8 |
| B06 | *Chgb* | Chromogranin B |
| B07 | *Cul2* | Cullin 2 |
| B08 | *Cxxc1* | CXXC finger 1 (PHD domain) |
| B09 | *Ddc* | Dopa decarboxylase |
| B10 | *Dlk1* | Delta-like 1 homolog (*Drosophila*) |
| B11 | *Drd2* | Dopamine receptor D2 |
| B12 | *Egln1* | EGL nine homolog 1 (*C. elegans*) |
| C01 | *Fbxo9* | F-box protein 9 |
| C02 | *Fgf13* | Fibroblast growth factor 13 |
| C03 | *Fn1* | Fibronectin 1 |
| C04 | *Gabbr2* | Gamma-aminobutyric acid (GABA) B receptor, 2 |
| C05 | *Gbe1* | Glucan (1,4-alpha-), branching enzyme 1 |
| C06 | *Gpr37* | G protein-coupled receptor 37 |
| C07 | *Gria3* | Glutamate receptor, ionotropic, AMPA3 (alpha 3) |
| C08 | *Hspa4* | Heat shock protein 4 |
| C09 | *Htr2a* | 5-hydroxytryptamine (serotonin) receptor 2A |
| C10 | *Kcnj6* | Potassium inwardly-rectifying channel, subfamily J, member 6 |
| C11 | *Lrrk2* | Leucine-rich repeat kinase 2 |
| C12 | *Mapk9* | Mitogen-activated protein kinase 9 |
| D01 | *Mapt* | Microtubule-associated protein tau |
| D02 | *Ncoa1* | Nuclear receptor coactivator 1 |
| D03 | *Nefl* | Neurofilament, light polypeptide |
| D04 | *Nfasc* | Neurofascin |
| D05 | *Nr4a2* | Nuclear receptor subfamily 4, group A, member 2 |
| D06 | *Nrxn3* | Neurexin III |
| D07 | *Nsf* | N-ethylmaleimide sensitive fusion protein |
| D08 | *Nsg1* | Neuron specific gene family member 1 |
| D09 | *Ntrk2* | Neurotrophic tyrosine kinase, receptor, type 2 |
| D10 | *Opa1* | Optic atrophy 1 homolog (human) |

| | | |
|---|---|---|
| D11 | *Pan2* | PAN2 polyA specific ribonuclease subunit homolog (*S. cerevisiae*) |
| D12 | *Park2* | Parkinson disease (autosomal recessive, juvenile) 2, parkin |
| E01 | *Park7* | Parkinson disease (autosomal recessive, early onset) 7 |
| E02 | *Pink1* | PTEN induced putative kinase 1 |
| E03 | *Ppid* | Peptidylprolyl isomerase D (cyclophilin D) |
| E04 | *Prdx2* | Peroxiredoxin 2 |
| E05 | *Psen2* | Presenilin 2 |
| E06 | *Pten* | Phosphatase and tensin homolog |
| E07 | *Rgs4* | Regulator of G-protein signaling 4 |
| E08 | *Rtn1* | Reticulon 1 |
| E09 | *S100b* | S100 protein, beta polypeptide, neural |
| E10 | *Sept5* | Septin 5 |
| E11 | *Skp1a* | S-phase kinase-associated protein 1A |
| E12 | *Slc18a2* | Solute carrier family 18 (vesicular monoamine), member 2 |
| F01 | *Slc25a4* | Solute carrier family 25 (mitochondrial carrier, adenine nucleotide translocator), member 4 |
| F02 | *Slc6a3* | Solute carrier family 6 (neurotransmitter transporter, dopamine), member 3 |
| F03 | *Slit1* | Slit homolog 1 (*Drosophila*) |
| F04 | *Snca* | Synuclein, alpha |
| F05 | *Spen* | SPEN homolog, transcriptional regulator (*Drosophila*) |
| F06 | *Srsf7* | Serine/arginine-rich splicing factor 7 |
| F07 | *Stub1* | STIP1 homology and U-Box containing protein 1 |
| F08 | *Sv2b* | Synaptic vesicle glycoprotein 2 b |
| F09 | *Syngr3* | Synaptogyrin 3 |
| F10 | *Syt1* | Synaptotagmin I |
| F11 | *Syt11* | Synaptotagmin XI |
| F12 | *Tcf7l2* | Transcription factor 7-like 2, T-cell specific, HMG-box |
| G01 | *Th* | Tyrosine hydroxylase |
| G02 | *Tpbg* | Trophoblast glycoprotein |
| G03 | *Uba1* | Ubiquitin-like modifier activating enzyme 1 |
| G04 | *Ubc* | Ubiquitin C |
| G05 | *Ube2i* | Ubiquitin-conjugating enzyme E2I |
| G06 | *Ube2k* | Ubiquitin-conjugating enzyme E2K (UBC1 homolog, yeast) |
| G07 | *Ube2l3* | Ubiquitin-conjugating enzyme E2L 3 |
| G08 | *Uchl1* | Ubiquitin carboxy-terminal hydrolase L1 |
| G09 | *Usp34* | Ubiquitin specific peptidase 34 |
| G10 | *Vamp1* | Vesicle-associated membrane protein 1 |
| G11 | *Vdac3* | Voltage-dependent anion channel 3 |
| G12 | *Ywhaz* | Tyrosine 3-monooxygenase/tryptophan 5-monooxygenase activation protein, zeta polypeptide |
| H01 | *Actb* | Actin, beta |
| H02 | *B2m* | Beta-2 microglobulin |
| H03 | *Gapdh* | Glyceraldehyde-3-phosphate dehydrogenase |
| H04 | *Gusb* | Glucuronidase, beta |
| H05 | *Hsp90ab1* | Heat shock protein 90 alpha (cytosolic), class B member 1 |
| H06 | MGDC | Mouse Genomic DNA Contamination |
| H07 | RTC | Reverse Transcription Control |
| H08 | RTC | Reverse Transcription Control |

| | | |
|---|---|---|
| H09 | RTC | Reverse Transcription Control |
| H10 | PPC | Positive PCR Control |
| H11 | PPC | Positive PCR Control |
| H12 | PPC | Positive PCR Control |

## SUPPLEMENTARY INFORMATIONS

**Figure S1A**: Full image of Figure 5 western blot for NAPE-PLD using anti-NAPE-PLD rabbit antibody, * indicates positive control for NAPE-PLD protein obtained by transient overexpression of the protein in Hek293 cells. The red rectangle portion of the gel was cropped and used in the main figure.

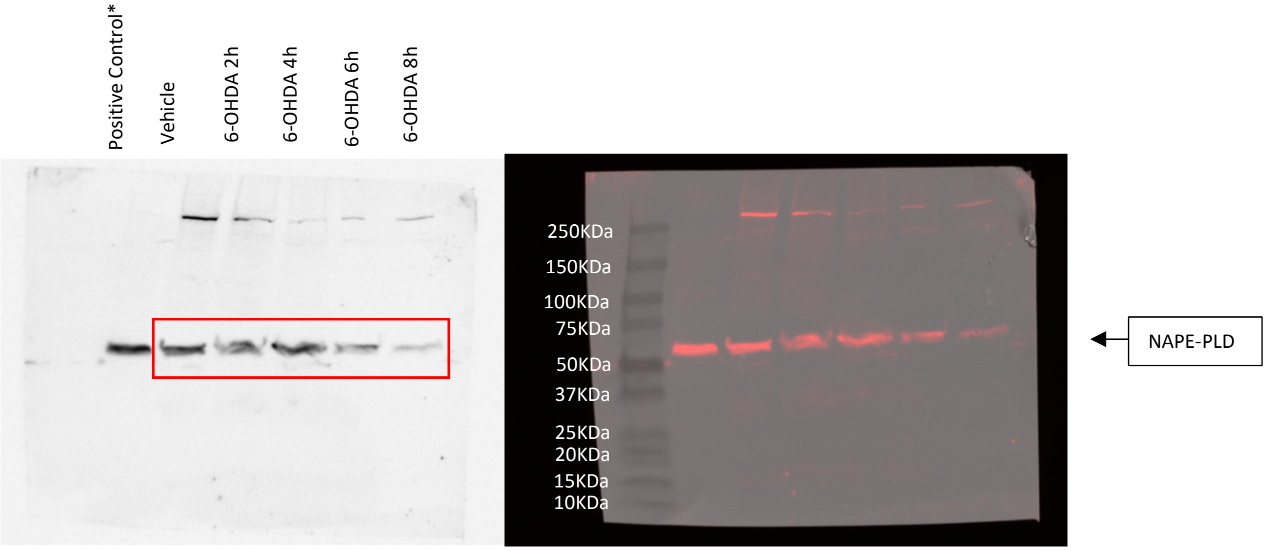


**Figure S1B**: Full image of Figure 5 western blot for GAPDH, the same membrane used in figure S1A was re-probed with anti-GAPDH antibody. The GAPDH signal was distinguished from the previous NAPE-PLD signal by their molecular weight (37KDa). The red rectangle portion of the gel was cropped and used in the main figure.

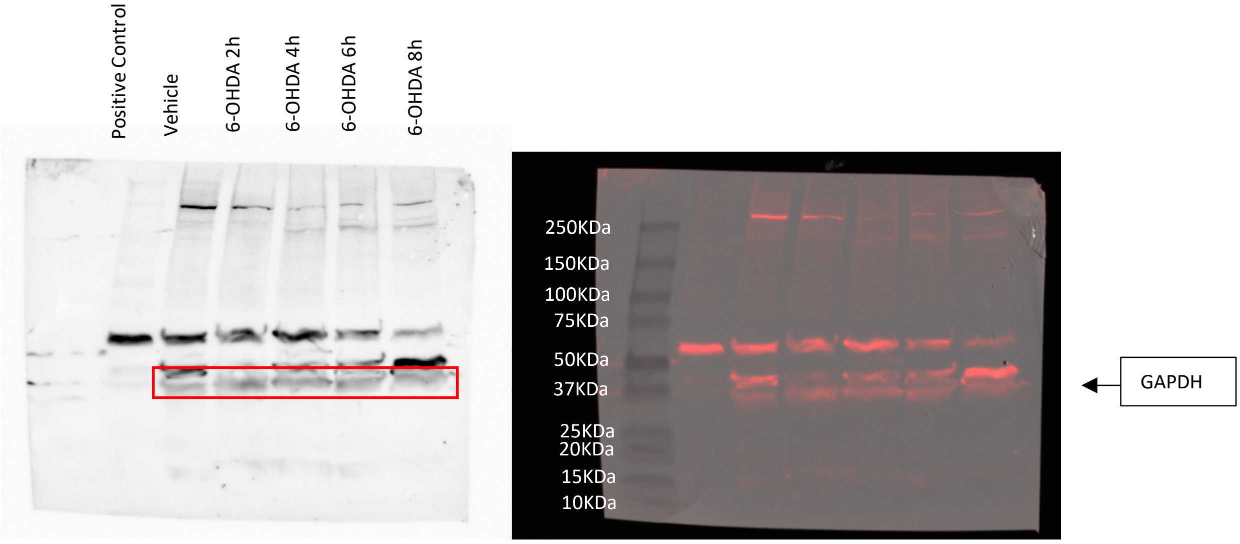

**Figure S2A**: Full gel image of Figure 6 western blot for NAPE-PLD using anti-NAPE-PLD rabbit antibody. The red rectangle portions of the gel were cropped and used as representative images in the main figure.

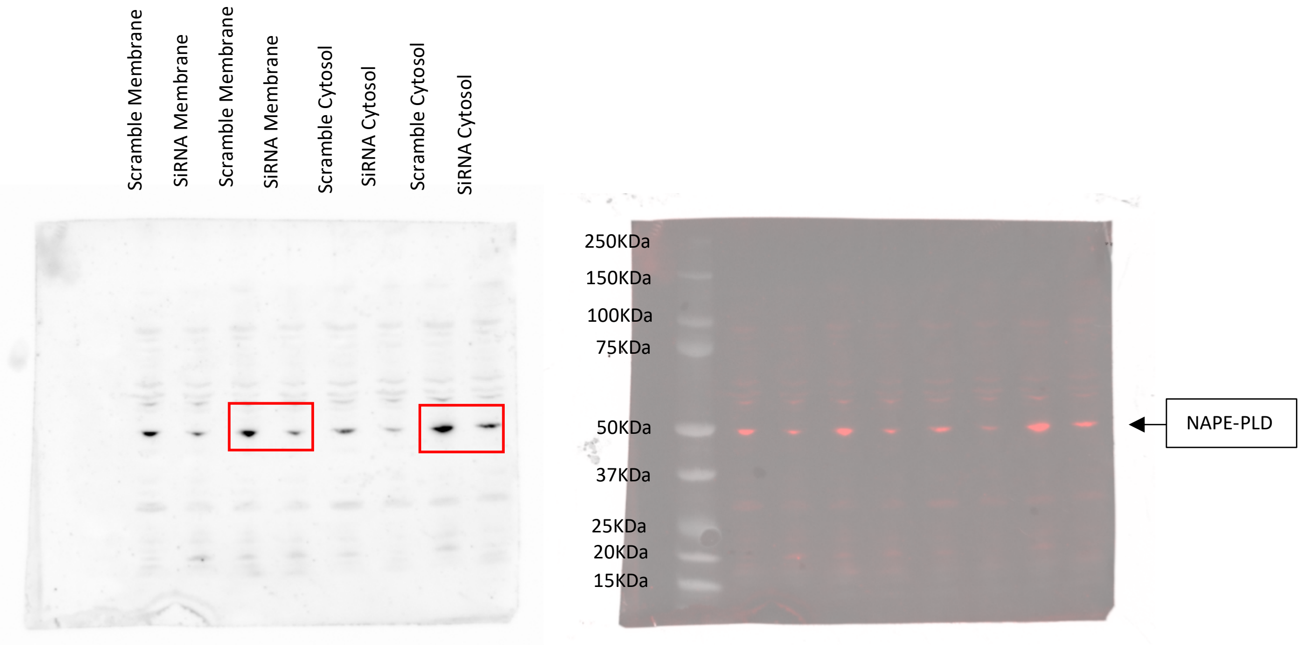


**Figure S2B**: Full image of Figure 6 western blot for GAPDH. The same membrane used in figure S2A was re-probed with anti-GAPDH antibody. The GAPDH signal was distinguished from the previous NAPE-PLD signal by their molecular weight. The red rectangle portion of the gel was cropped and used in the main figure.

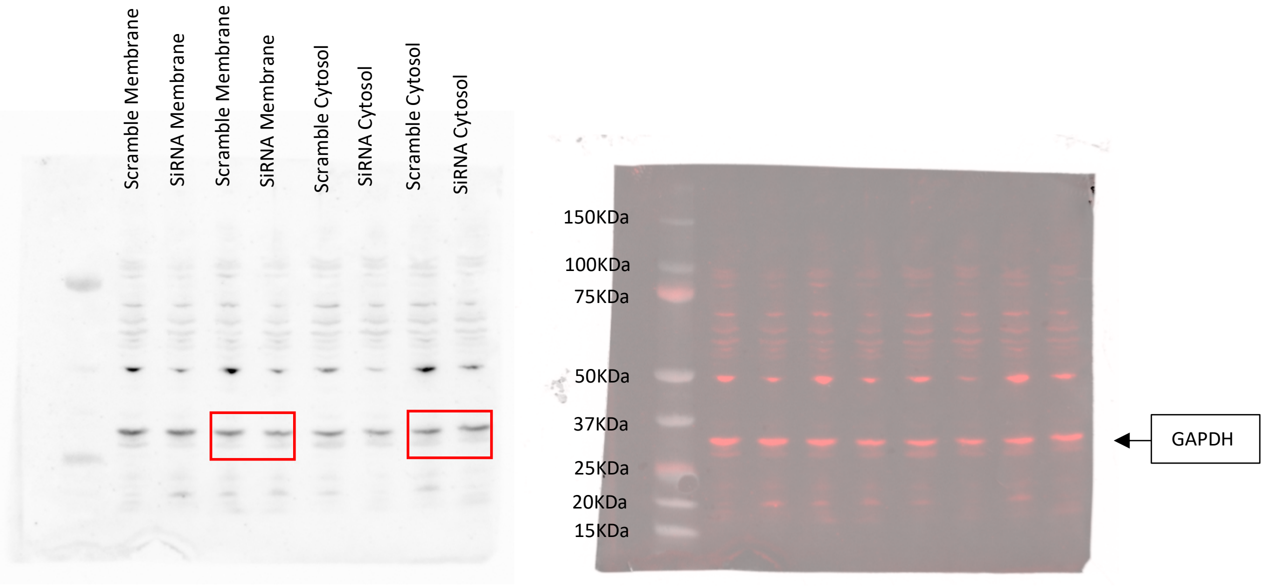

**Figure S3A**: Full gel image of Figure 8 western blot for Rac1-GTP using anti-Rac1-GTP antibody. The red rectangle portions of the gel were cropped and used as representative images in the main figure.

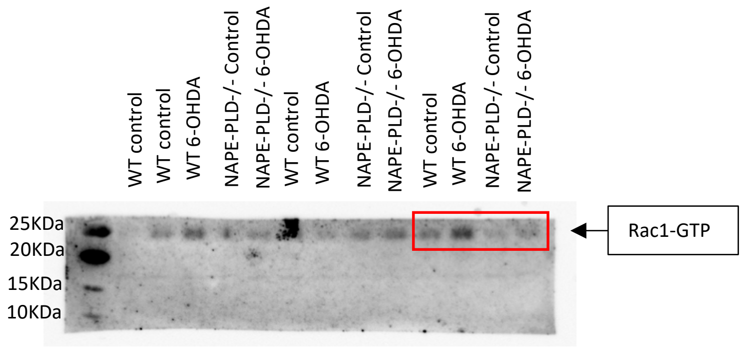


**Figure S3B**: Full gel image of Figure 8 western blot for GAPDH using anti-GAPDH rabbit antibody. The red rectangle portions of the gel were cropped and used as representative images in the main figure.

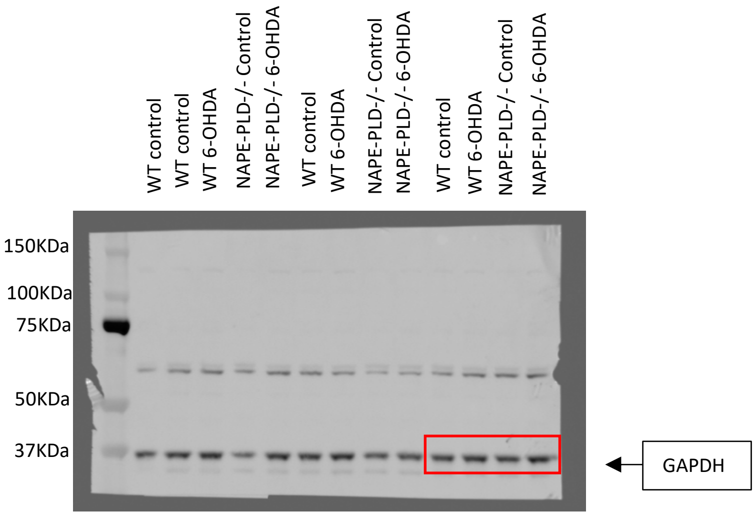


**Figure S3C**: Full gel image of Figure 8 western blot for Rac1 using anti-Rac1 antibody. The red rectangle portions of the gel were cropped and used as representative images in the main figure.

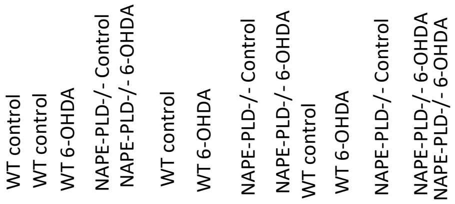

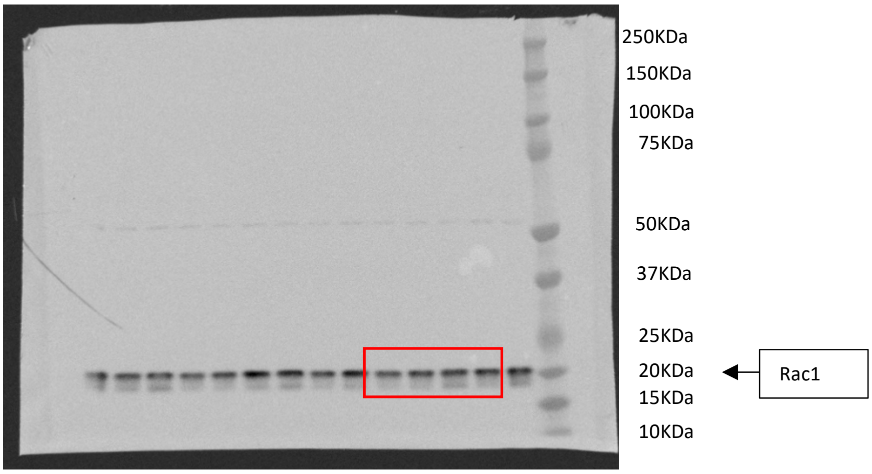


**Figure S3D**: Full gel image of Figure 8 western blot for GAPDH using anti-GAPDH rabbit antibody. The red rectangle portions of the gel were cropped and used as representative images in the main figure.

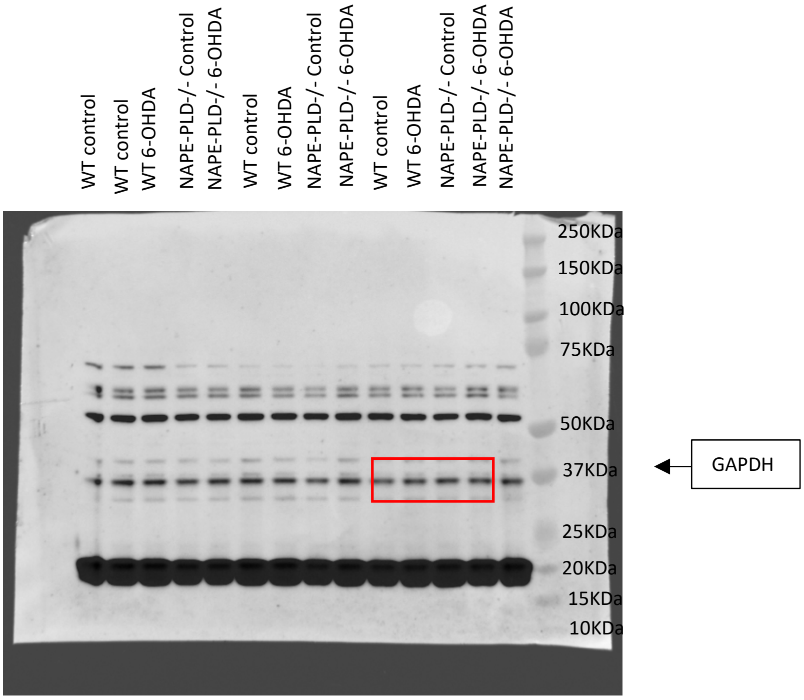

Supplement: Supplementary file 1 — Supplementary info [file 41598_2019_51799_MOESM1_ESM.pdf]
